# *Leishmania donovani* elongator protein Elp3a plays a crucial role in modulating the parasite response to genotoxic stress

**DOI:** 10.1128/spectrum.02439-25

**Published:** 2025-12-05

**Authors:** Arushi Khanna, Shilpa Rohra, Swati Saha

**Affiliations:** 1 Department of Microbiology, University of Delhi South Campus 93081, New Delhi, India; CSIR-Institute of Microbial Technology, Chandigarh, India

**Keywords:** *Leishmania donovani*, trypanosome, protozoan parasite, elongator, Elp3a, genotoxic stress

## Abstract

The eukaryotic elongator complex comprises six proteins Elp1–Elp6, with Elp3 being the catalytic subunit. In trypanosomatids, only Elp3 has been identifiable, and interestingly, these organisms have two Elp3 orthologs: Elp3a and Elp3b. This study was undertaken to examine the role of Elp3a in the trypanosomatid *Leishmania donovani*. The *elp3a* gene was successfully eliminated, with the insect form as well as mammalian form of the parasite surviving and propagating normally in its absence, signifying its non-essential nature under usual growth conditions. In examining the response of LdElp3a-depleted parasites to genotoxic agents, we found no behavioral differences in response to UV and camptothecin, but the parasites exhibited higher tolerance to methyl methane sulfonate and chronic hydroxyurea treatment. This was not due to a hyperactive DNA damage response in Elp3a-depleted cells; rather, these cells suffered less DNA damage *per se*. In looking for possible mechanisms by which Elp3a-depleted parasites were being protected from the effects of these genotoxic agents, we considered previous findings in yeast where Elp3 (as part of the Elp1–6 complex) had been found to play a role in the modification of transfer RNA uridines at the wobble position of anticodons, thus promoting translation efficiency. However, while yeast *elp3* mutants displayed a downregulation in translation of proteins carrying adenine residues at the wobble position of their codons, our experimental results suggest that this mechanism of regulation does not exist in *Leishmania* species, perhaps in synchrony with the fact that the Elp1–6 holocomplex itself does not seem to be present in these parasites.

**IMPORTANCE:**

Leishmaniases are a group of diseases endemic to 90 countries, putting over a billion people at risk of infection. No vaccines are available against this digenetic parasite that cycles between the insect host sandfly and the mammalian host. While drugs are available to treat the disease, their high costs and toxic side effects combined with the problems of emerging drug resistance continue to give a thrust to research in the areas of their cellular biology. The Elp3 protein in eukaryotes is known to modulate a myriad of processes. This study aims to investigate the role of the *Leishmania donovani* Elp3a protein. We find the protein is not essential for parasite survival but plays a role in moderating the parasite response to certain genotoxic stresses. Thus, Elp3a regulates one or more processes that help the parasite survive in inhospitable environments that trigger DNA damage.

## INTRODUCTION

Elongator was originally identified as a multi-subunit complex that co-purified with RNA pol II, and as its association with RNA Pol II depended on the phosphorylation of the RNA pol II C-terminal domain, it was believed to be a component of transcriptional elongation machinery in budding yeast (1). The catalytic subunit of Elongator, Elp3, harbors a domain typically found in the GCN5-related N-acetyltransferase (GNAT)-family of histone acetyltransferases: lysine (K) acetyltransferase domain (KAT). Wittschieben et al. (2) proposed that the association of a histone acetyltransferase activity with RNA PolII during transcriptional elongation might facilitate the process through acetylation of nucleosomal histones, leading to a more open chromatin. Subsequently, Winkler et al. (3) demonstrated that *elp3a*-nulls exhibited lowered levels of H3 and H4 acetylation in budding yeast, and this GNAT-family histone acetyltransferase was also found to mediate H3 acetylation in human cells (4), reinforcing the suggestion that it promotes transcriptional elongation. *Saccharomyces cerevisiae elp3*-nulls exhibited shortened nucleosomal DNA lengths, implying that Elp3-mediated histone acetylation may play a role in nucleosomal assembly as well (5). Found in all three domains of life, the Elongator complex is now known to be involved in a myriad of cellular processes, including replication elongation, protein acetylation, DNA damage response, and transfer RNA (tRNA) modifications (6–8).

While Elongator and its components Elp1-Elp6 have been extensively researched in budding and fission yeast as well as metazoans, only limited information is available in trypanosomatids. Genome sequence annotations have identified only Elp3 orthologs (9, 10). Interestingly, trypanosomatids carry two *elp3* genes, designated *elp3a* and *elp3b*, whose sequences are divergent from those of Elp3 proteins of other eukaryotes (11). Alsford and Horn demonstrated that the *Trypanosoma brucei* Elp3b localized with the nucleolus, playing a role in controlling the levels of rDNA transcription. The same study found the TbElp3a protein to localize to the nuclear periphery, but although an *elp3a*-null was successfully created, no differential phenotypes (compared to wild-type parasites) were reported (11).

*Leishmania* species are trypanosomatids that cause the group of diseases called leishmaniases, endemic to 90 countries. Of the three forms of the disease (cutaneous, mucocutaneous, and visceral), visceral leishmaniasis (VL) (commonly called kala-azar) can be fatal if not treated in a timely fashion. No vaccine is available, and current drugs are expensive and have toxic side effects, with treatment regimens being lengthy and complex. These problems are compounded by emerging drug resistance and risks associated with *Leishmania*-HIV co-infections as well as threats from post-kala-azar dermal leishmaniasis (12). With more than a billion people at risk of infection (https://www.who.int/news-room/fact-sheets/detail/leishmaniasis), several groups are engaged in studying this unicellular parasite’s cellular processes in the hope of identifying potential sites for therapeutic intervention. Annotation of the genome sequence of *Leishmania donovani*, the causative pathogen of VL in the Indian subcontinent, revealed that this trypanosomatid too harbors two *elp3* genes (13), and this study investigates the role of Elp3a in the parasite.

*Leishmania* species are digenetic, shuttling between the mammalian host where they exist intracellularly in macrophages as non-motile amastigotes and the insect host (sandfly) where they are transformed and exist extracellularly in the midgut as flagellate promastigotes, initially as non-infective procyclics, then differentiating into nectomonads and subsequently leptomonads, before developing into infective metacyclics which migrate to the salivary glands (14). The metacyclics are released into the mammalian bloodstream with the insect bite. The data presented here indicate that *L. donovani elp3a* is not essential for cell survival in either the promastigote or amastigote stage of the parasite. However, LdElp3a moderates the parasite’s response to certain agents known to induce genotoxic stress. The implications of these findings are discussed.

## MATERIALS AND METHODS

### *Leishmania* cultures and manipulations

*L. donovani* 1S (Ld1S) promastigotes were grown in M199 (Lonza, Switzerland and Sigma Aldrich, USA) as described (15). Growth of *Leishmania* parasites in *in vitro* cultures was analyzed as described (16). Cells were synchronized using hydroxyurea (HU) as previously (17). Cells were evaluated for cell cycle progression by flow cytometry as described (18). Whole cell extracts were made using the M-PER reagent (Thermo, USA).

### Cloning of *L. donovani elp3a* gene and expression of LdElp3a in *Leishmania* promastigotes

The *elp3a* gene was amplified with adaptor-primers Elp3a-F: 5′- TGGCCGTGCCGGCCACCTTATGTCCTCTGATAGC-3′ and Elp3a-R: 5′- TGGCCGGCACGGCCCAGAGTGCGCGCCGCT-3′ and Phu DNA polymerase, using Ld1S genomic DNA as template. The ~2.3 kb amplicon obtained was cloned into pUC19 for sequencing. For expression in fusion with FLAG, the *elp3a* gene was released from its pUC clone using SfiI restriction digestion and subcloned into the same site in the pXG-FLAG vector (17). To express the tagged protein in *Leishmania*, the plasmid was transfected into promastigotes as earlier (19) and clonals selected for using G418. Expression of the tagged protein in clonals was checked by Western blot analysis of clonal extracts, and clonals expressing the protein robustly were analyzed microscopically for subcellular localization.

### Immunofluorescence analysis

Immunofluorescence analysis was carried out as described earlier (20). Briefly, *Leishmania* promastigotes were fixed in 2% paraformaldehyde, cells spread on poly-lysine coated coverslips, permeabilized with 0.1% Triton X-100, blocked with chicken serum (10%), incubated with primary antibody (1:100 FLAG antibody: Cat. no. F1804, Sigma Aldrich, USA), washed and incubated with Texas Red-labeled secondary antibody (1:100, Jackson ImmunoResearch Laboratories, USA), and washed and mounted in Vectashield antifade mounting medium carrying DAPI (VectorLabs, USA). Cells were viewed using a 100× (in oil) objective in a Leica TCS SP8 confocal microscope. Z-stack images were captured and analyzed using Leica LAS AF software.

### Construction of donor plasmids

To create *elp3a*-nulls, two donor plasmids were constructed: one carrying the *hyg^r^* cassette and the other carrying the *neo^r^* cassette. The sequence immediately upstream of the *elp3a* gene (5′ flank) was amplified using primers Elp3a-5’Fl-F: 5′-TCGCGGCCGCCTCGAGGTATCGCACTTCGTC-3′ and Elp3a-5′Fl-R: 5′-TCGCGGCCGCGACGGGCACACCCC AAGAGCA-3′, while the sequence immediately downstream of the *elp3a* gene (3′ flank) was amplified using primers Elp3a-3′Fl-F: 5-TCCCTAGGGTAAATCGCACAACTCTTCC-3′ and Elp3a-3′Fl-R: 5′-TTCCTAGGGATATCAAGTCGAAGCTGCAA-3′. The 5′ flank sequence was cloned into the NotI site of vectors pLEXSY-eGFP-neo3 (Jena Bioscience, Germany) and pLEXSY-eGFP-hyg (21), and the 3′ flank sequence was cloned into the SpeI site of the constructs so generated. Thus, donor plasmids pElp3a-KO/neo and pElp3a-KO/hyg were created, with the drug resistance cassette in both donor constructs being flanked by appropriate donor sequences.

### Generation of *elp3a* knockout and rescue lines

To create *elp3a^−/+^*, the donor cassette released from pElp3a-KO/neo using XhoI-EcoRV digestion was transfected into logarithmically growing *Leishmania* promastigotes and clonals selected for as earlier (19). Briefly, transfection mixes were plated on semi-solid M199 carrying G418 (50 µg/mL), and colonies visible 10–14 days later were expanded step-wise. Genomic DNA of these clonals was screened for homologous recombination (HR) by PCRs across the deletion junctions. To create *elp3a^−/−^*, the donor cassette released from pElp3a-KO/hyg using EcoRV digestion was transfected into logarithmically growing *elp3a^−/+^* promastigotes and transfectant clonals selected for using G418 (50 µg/mL) and hygromycin (16 µg/mL). Genomic DNA isolated from these clonals was similarly screened for HR.

To create the *elp3a^−/−^::*Elp3a^+^ rescue line, the *elp3a* gene was cloned into the SfiI site of pXG-FLAG (bleo) vector (19) and the plasmid pXG/Elp3a-FLAG (bleo) transfected into logarithmically growing *elp3a^−/−^* promastigotes. Transfectant colonies were selected for using bleomycin (2.5 µg/mL), G418, and hygromycin. The clonals were screened by Western blot analysis of isolated whole cell lysates.

### Induction of genotoxic stress in *Leishmania* promastigotes

The effect of UV irradiation on promastigotes was evaluated as described earlier (19). Briefly, cultures were initiated at a density of 1 × 10^5^ cells/mL from mid-log phase cells 48 hours prior to UV irradiation. At the time of UV exposure, 6 × 10^5^ cells/mL were dispensed in the wells of a six-well cluster dish (1 mL per well; four wells per cell type), exposed to UV (254 nm) with a hand-held UV torch (of intensity 400 μW/cm^2^) held 1 cm away from the cells, and allowed to recover under white light after adding equal volume of fresh medium. Cells were counted every 24 hours.

The effects of camptothecin (CPT)-induced and methyl methane sulfonate (MMS)-induced genotoxic stress on *Leishmania* promastigotes (wild-type and mutant) were assessed by seeding promastigote cultures at 1 × 10^6^ cells/mL from stationary phase cultures and adding CPT (0–25 µM) or MMS (0%–0.1%) to the cultures on Day 3 (when cultures were at 7–9 × 10^6^ cells/mL) before continuing incubation at 26°C. Cells were counted every 24 hours. The experiment was conducted thrice, and values plotted are the average of three experiments. Error bars show standard deviation.

The effect of hydroxyurea (HU)-induced chronic stress was investigated by initiating cultures at 1 × 10^6^ cells/mL from stationary phase cultures and adding 0.5 mM or 1 mM HU 48 hours later. Following a 24-hour incubation with HU, the drug was washed off and cells replenished with fresh HU-free medium, before continuing growth for another 72 hours. Cells were counted every 24 hours. The experiment was conducted thrice, and values plotted are the average of three experiments. Error bars show standard deviation.

### TUNEL assay

Terminal deoxynucleotidyl transferase dUTP Nick-End Labeling (TUNEL) assay was performed using the Dead End Fluorometric TUNEL System (Promega), as described earlier (22). Briefly, logarithmically growing cells were incubated in M199 medium carrying HU (1 mM) for 24 hours at 26°C, before collecting them by low-speed centrifugation (1,448 g for 3 minutes), washing off the drug with 1× phosphate-buffered saline (PBS), and fixing the cells. For cells analyzed 6.5 hours after release from drug, after washing off the drug with 1× PBS, the cells were replenished with fresh M199 and incubated for a further 6.5 hours at 26°C, before fixing. Fixed cells were spread on poly-lysine coated coverslips, permeabilized with 0.2% Triton X-100, washed, and incubated in tailing reaction mix (containing fluorescein-labeled dUTP) for an hour before quenching the reaction. The coverslips were mounted in DAPI-containing anti-fade medium. The cells were observed microscopically for fluorescein-labeled nuclei, with a Leica TCS SP8 confocal microscope using 100× (in oil) objective, by excitation at 488 nm and detecting fluorescence emission at 510 nm. Labeled nuclei were analyzed using Leica application suite X (LAS X) software. The experiment was performed thrice with comparable results.

### Chromatin isolations

Isolation of soluble and DNA-bound fractions of cell lysates was carried out as described earlier (19). Briefly, 5 × 10^7^ cells were collected by centrifugation, washed with 1× PBS, resuspended in 75 µL of lysis buffer (10 mM Tris-HCl pH 7.4, 3 mM MgCl_2_, 300 mM sucrose, 100 mM NaCl, 0.1% Triton X-100, 50 mM sodium butyrate and protease inhibitors), incubated with rotation at 4°C for 10 minutes, and the supernatant collected by centrifugation at 1,300 *g* for 5 minutes at 4°C. The pellet was subjected to a second round of similar treatment, and the supernatants of both rounds were clarified by high-speed centrifugation, to obtain soluble protein fractions (S1 and S2). To isolate DNA-associated fractions, the pellet obtained after the second lysis step above was treated with 50 U of DNAase I (Thermo Fisher Scientific) at 25°C for 40 minutes, the reaction centrifuged at 1,700 *g* for 5 minutes at 4°C, and supernatant collected as the DNA-bound protein fraction (S3). The pellet was subjected to a second round of similar DNAase I treatment to obtain the DNA-bound protein fraction S4.

### Macrophage infections

Infections of J774A.1 macrophages were carried out as described earlier (21). Intracellular parasite load was determined by counting the DAPI-stained nuclei of parasites in Z-stack images of infected macrophages, captured with confocal microscopy. To carry out infections of THP1 cells, 1 × 10^6^ THP1 monocytes were plated on poly-lysine coated coverslips in the wells of a six-well cluster dish, in complete RPMI 1640 medium carrying phorbol 12-myristate-13-acetate (20 nM; Sigma Aldrich, USA) to induce differentiation to macrophages. After 24 hours, the cells were washed with 1× PBS and replenished with complete RPMI medium, followed by a 24-hour recovery period before setting up infections. Infections were set up by adding ~2×10^7^ metacyclics to each well in serum-free RPMI medium. After 10 hours, the non-internalized parasites were removed, the wells washed with 1× PBS, and cells refed with 2 mL of complete medium. Slides were prepared at various times thereafter and analyzed for intracellular parasite load as above. Confocal microscopy images were analyzed using LAS X software. J774A.1 and THP1 infections were each performed thrice, and values plotted are the average of the three experiments. Error bars depict standard deviation. Statistical significance was determined using student’s two-tailed *t*-test.

## RESULTS

### *L. donovani* Elp3a protein is constitutively nuclear

**Fig 1:**
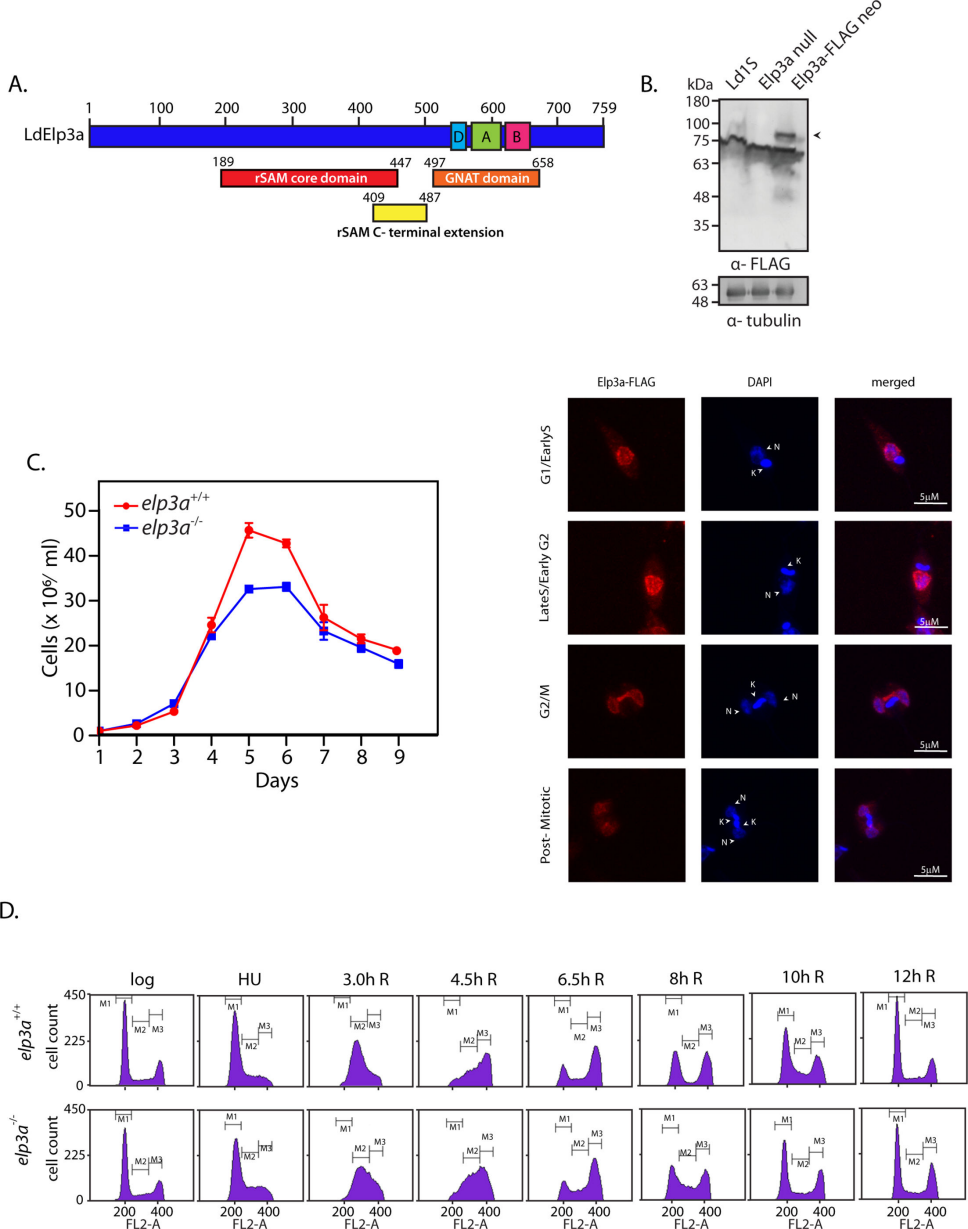
*elp3a* is not essential for survival of *Leishmania* promastigotes. (**A**) Conserved domains identified in LdElp3a. Boxes demarcate the relevant domains. Numbers indicate the positions of the starting and ending amino acids of the domains. (**B**) Analysis of subcellular localization of LdElp3a-FLAG. Upper panel: Western blot analysis of whole cell lysates isolated from transfectant promastigotes using anti-FLAG antibodies (1:2,500 dil). Tubulin served as loading control. Arrowhead indicates ~85 kDa Elp3a-FLAG protein. Full-length uncropped blots in Fig. S6. Lower panel: microscopic analysis of transfectant promastigotes using immunofluorescence with anti-FLAG antibodies. DAPI stained both the nuclear (N) and kinetoplast (K) compartments. Kinetoplast morphology and segregation pattern was used as cell cycle stage marker. G1/early S: roundish/short rod-like kinetoplast, single nucleus (1N, 1K). Late S/early G2: one elongated kinetoplast, single nucleus (1N,1K). G2/M: one kinetoplast, two nuclei (2N,1K). Post-mitosis: two kinetoplasts, two nuclei (2N, 2K). Magnification bar: 5 µm. (**C**) Analysis of growth of *elp3a*^−/−^ promastigotes. Cultures were initiated from stationary phase cultures. The experiment was done thrice with technical replicates in each experiment. Values plotted are average of three experiments, and error bars indicate standard deviation. Raw data excel sheets in Fig. S7. (**D**) Analysis of *elp3a*^−/−^ cell cycle progression. Cells were synchronized at G1/S boundary with 5 mM HU and then released into fresh drug-free medium. Sampling time-points are indicated above the histogram frames. “R” refers to hours after release. Cells in G1, S, and G2M are gated as M1, M2, and M3, respectively. The experiment was done thrice, with comparable results, and one dataset is shown here. The gating strategy is shown in Fig. S15.

 In S. *cerevisiae* and metazoans, the Elp3 protein is part of a six-subunit Elongator complex, implicated to play a role in nuclear as well as cytosolic processes. The present study was initiated with an analysis of the subcellular localization of the protein, to gage its possible roles for further investigations. For this, the *elp3a* gene was amplified off Ld1S genomic DNA using end-primers designed against LdBPK_160250.1 (sequence obtained from the Tritryp DB ; [23], www.tritrypdb.org) as the genome sequence of Ld1S is not available. The ~2.3 kb *elp3a* amplicon was cloned and sequenced (GenBank Accession PX023932), and analysis of the derived amino acid sequence by Clustal Omega (24) revealed that LdElp3a protein shared 60%–99% identity over 90%–100% coverage with other trypanosomatid Elp3a proteins (Fig. S1) and 36%–38% identity over 82%–87% coverage with the bacterial, yeast, and human Elp3 proteins (Fig. S2). LdElp3 carried an N-terminal radical S-adenosyl methionine (rSAM) binding domain (residues 189–447) and a C-terminal KAT domain (residues 497–658; Fig. 1A). This domain structure is typical of Elp3 proteins across all three domains of life, and considering the range of roles Elp3 has been implicated in, the roles of the two domains have been much discussed. The KAT domain carries the A, B, and D motifs that are conserved in GNAT-family histone acetyltransferases, with the A and D motifs believed to be mediating the binding of acetyl-CoA (25, 26). While early studies proposed the KAT domain to play a role in histone acetylations and thus the modulation of transcription (1, 3), later findings have underscored the importance of this domain of Elp3 in mediating tRNA modifications (27). Evidence points to Elp3 catalyzing the carboxymethylation of tRNA anticodon wobble uridines at the fifth position of the pyrimidine ring, where the KAT domain of Elp3 transfers a carboxymethyl group from acetyl-CoA to the pyrimidine ring, and this is followed by further modifications by other enzymes (28). The rSAM domain of Elp3 is proposed to facilitate the transfer of the methyl group from SAM to the carboxymethyl group by a tRNA methyltransferase (27). In attempting to acquire the 3D structure of LdElp3a by homology-based modeling using the Phyre2.0 engine, we found that the 759 amino acid LdElp3a protein demonstrated maximum similarity with the *Dehalococcoides mccartyi* Elp3 (DmElp3; 42% identity over 62% coverage with 100% confidence against PDB ID:5L7J; Fig. S3). The 2.15 Å crystal structure of DmElp3 revealed that tRNA binds to the interface of the N-terminal rSAM domain and C-terminal KAT domain (29). DmElp3 demonstrates tRNA wobble uridine modification activity *in vitro* (28). In eukaryotes, however, wobble uridine modifications are catalyzed by Elp3 as part of the Elongator holo-complex. Thus far, there is no evidence of the existence of an Elongator complex in *L. donovani* or any other trypanosomatid, and in-depth studies need to be carried out to determine if trypanosomatid Elp3 proteins possess tRNA modifying ability *in vitro* or *in vivo*.

 To gain insight into whether LdElp3a is involved in one or more nuclear and/or cytoplasmic processes, we examined the subcellular localization of LdElp3a by expressing the FLAG-tagged protein in *Leishmania* promastigotes and observing transfectant promastigotes microscopically using immunofluorescence, as described (Materials and Methods). We found that LdElp3a-FLAG was predominantly nuclear throughout the cell cycle, also being detected in the cytosol in G1 and S phases (Fig. 1B). These findings were in keeping with earlier findings with TbElp3a where the protein was primarily nuclear (11, 30). Based on the distribution pattern of LdElp3a, we surmised that its role may most likely be nuclear.

### The *elp3a* gene is not essential for parasite survival

* *To identify the cellular roles of LdElp3a, we took the course of creating *elp3a* genomic knockouts and analyzing the phenotypes obtained. Genomic knockouts were created step-wise, replacing one allele at a time with drug resistance cassettes using HR in promastigote parasites (detailed in Materials and Methods). The first allele in Ld1S was replaced with a *neo^r^* cassette to create the heterozygous knockout *elp3a* ^–/+^, and the legitimacy of recombination at both ends was verified by PCRs across the deletion junctions (Fig. S4A). The second allele was replaced in the *elp3a* ^–/+^ line, with a *hyg^r^* cassette, and recombination at both ends verified by PCRs across the deletion junctions (Fig. S4B). The successful creation of *elp3a*-null (*elp3a* ^–/–^), confirmed by *elp3a*-specific PCR (Fig. S4B), underscored the fact that this gene is not essential for promastigote cell survival. Analysis of growth of *elp3a*-null promastigotes revealed that loss of *elp3a* did not impact early growth and growth in the logarithmic phase, but cells reached stationary phase at a lower cell density than usual, while maintaining the same length of stationary phase before entering the death phase (Fig. 1C). When cell cycle progression pattern was examined by synchronizing cells at the G1/S transition using an 8-hour HU (5 mM) block before releasing cells into S phase and monitoring their navigation across S, G2/M, and back into G1, we found *elp3a*-nulls behaved like wild-type cells, which was not surprising considering their similar growth patterns (Fig. 1D). In the absence of any major alterations in growth and cell cycle progression patterns, we concluded that LdElp3a did not modulate global gene expression in promastigotes under normal *in vitro* growth conditions, or if it did, its role must be redundant with one or more other cellular players.  

Considering the fact that *elp3a*-nulls reached stationary phase at a lower cell density than wild-type cells and that cells in stationary phase are metacyclic promastigotes which are the only stage of promastigotes that have the capability to infect the mammalian host cells, we investigated if the loss of *elp3a* compromised the parasite’s ability to infect host macrophages and/or propagate in them. Infection of macrophages with *Leishmania* parasites is carried out using murine macrophages or monocyte-derived human macrophages. Infection of murine macrophage J774A1 cells with *L. donovani* parasites is accompanied by production of pro-inflammatory cytokines IL-12 and TNF-α as well as the production of reactive nitrogen species (RNS) like nitric oxide, which would restrict parasite survival and proliferation. The parasite circumvents these hazards using its own range of protective mechanisms and modulates the host immune response to favor the production of anti-inflammatory cytokines which allow the parasite to survive (31). The establishment of infection in these macrophages is thus a battle between the host immune response and the parasite’s reaction to it. In infection experiments set up under the conditions detailed in Materials and Methods, the intracellular parasite load drops sharply within 24 hours after infection, before being maintained at more or less steady levels (21, 22). Human monocyte THP1 cells that are differentiated into macrophages behave somewhat differently upon *L. donovani* infection, producing the anti-inflammatory cytokine IL-10 rather than TNF-α, favoring an environment where the parasite thrives (32). Thus, the intracellular parasite load steadily increases with time (33, 34). We examined parasite survival/proliferation in both cell lines: J774A1 and THP1, to determine the impact of Elp3a elimination on the parasite response to the host environment.

**Fig 2:**
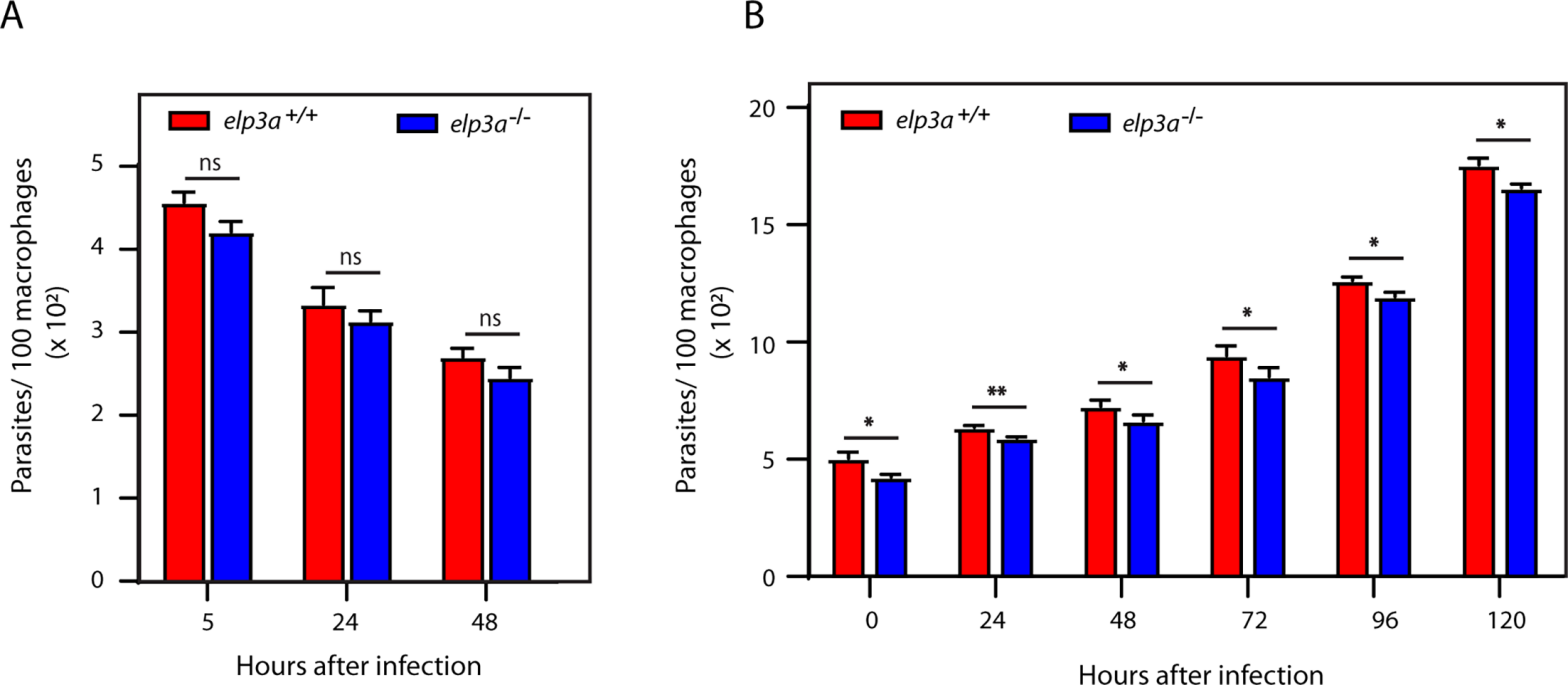
Deletion of *elp3a* has no impact on survival of the parasite within the mammalian host cell. (**A**) Analysis of murine macrophages (J774A.1) infected with *Leishmania* metacyclics. (**B**) Analysis of human macrophages (THP1 cells) infected with *Leishmania* metacyclics. Intracellular parasites were scored by mounting the cells in DAPI-containing medium and capturing Z-stack images using a confocal microscope, followed by image analysis using LAS X software. Both experiments were performed thrice, and average values are plotted in the bar graphs. Error bars denote standard deviation. Statistical significance was determined using the student’s *t*-test. *: *P* < 0.05, **: *P* < 0.005, ns: not significant. Raw data Excel sheets in Fig. S8.

Accordingly, J774A1 cells were infected with *Leishmania* metacyclics for 5 hours, before washing off non-internalized parasites and continuing incubation at 37°C in 5% CO_2_. The infected macrophages were analyzed microscopically at various time-points thereafter (as described in Materials and Methods), and intracellular parasites were scored by counting the DAPI-stained nuclei. We observed that the intracellular parasite load of *elp3a*-nulls was comparable to that of wild-type parasites at all time-points, signifying that deletion of *elp3a* had no impact on the parasite’s ability to infect the host macrophages and propagate within them (Fig. 2A). A similar analysis carried out with differentiated THP1 cells (detailed in Materials and Methods) revealed that both *elp3a^+/+^* and *elp3a^−/−^* parasites could infect and propagate within the macrophages comparably (Fig. 2B), suggesting that LdElp3a has no major role in parasite growth and survival within the mammalian host cells. In both experiments, the progression pattern of wild-type intracellular parasite load within the host cells was comparable to earlier reports (22, 33).

### Impact of genotoxic agents on Elp3a-depleted parasites

Since Elp3a-depleted promastigotes did not show any significant difference in *in vitro* growth and cell cycle progression patterns under normal conditions, nor did the deletion of *elp3a* have any significant impact on intracellular parasite survival in the mammalian host cells, we speculated that the protein might be involved in pathways activated under specific conditions like stress triggered by DNA damage-inducing agents. The impact of *elp3a* deletion on the cellular response to genotoxic stress was studied by exposing *elp3a*^−/−^ parasites to four genotoxic agents: UV irradiation, CPT, MMS, and HU (chronic treatment).

**Fig 3:**
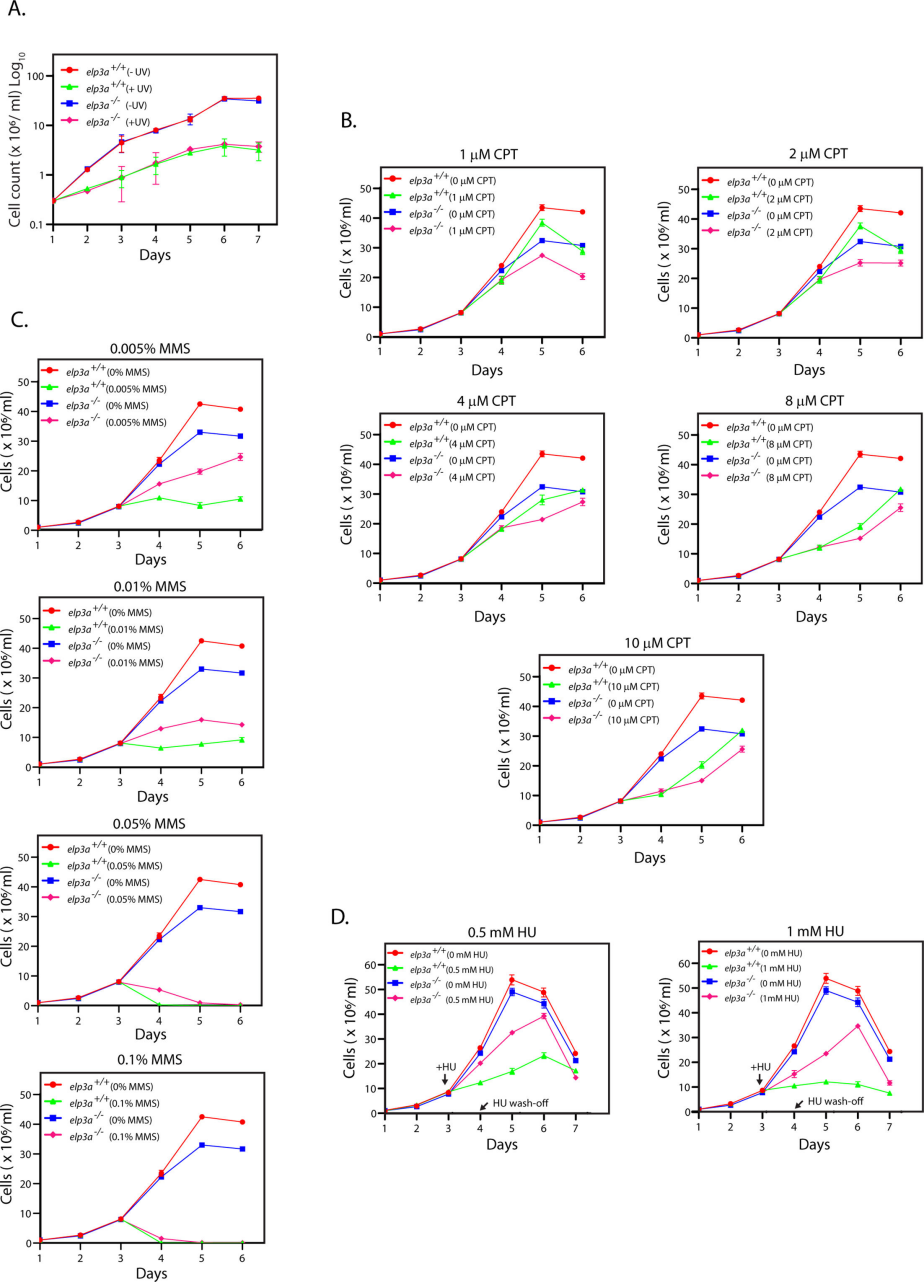
Effect of exposure to genotoxic agents on *elp3a*-nulls. (**A**) Effect of LdElp3a depletion on the parasite’s response to UV exposure. Cells were exposed to UV irradiation for 30 s (254 nm, with a hand-held torch of intensity 400 µW/cm^2^). Cells were counted every 24 hours thereafter. The experiment was performed twice, and average values are plotted in the graph. Error bars depict standard deviation. Raw data excel sheets are in Fig. S9. (**B**) Effect of LdElp3a depletion on the parasite’s response to CPT exposure. Cultures were seeded from stationary phase cultures and CPT (0–10 µM) added after 48 hours. Cells were counted every 24 hours. The experiment was performed thrice, and average values are plotted in the graphs. Error bars denote standard deviation. The data for each CPT concentration tested is presented in separate panels for easier viewing; the 0 µM graph lines are therefore identical across all panels. Raw data Excel sheets in Fig. S10. (**C**) Effect of LdElp3a depletion on the parasite’s response to MMS exposure. Cultures were seeded from stationary phase cultures and MMS (0%–0.1%) added after 48 hours. Cells were counted every 24 hours. The experiment was performed thrice, and average values are plotted in the graphs. Error bars denote standard deviation. The data for each MMS concentration tested is presented in separate panels for easier viewing; the 0 % graph lines are therefore identical across all panels. Raw data excel sheets in Fig. S11. (**D**) Effect of LdElp3a depletion on the parasite’s response to HU exposure. Cultures were seeded from stationary phase cultures, and HU (0, 0.5, and 1 mM) was added after 48 hours. Cells were incubated in the HU for 24 hours before washing off the drug and continuing incubation in fresh drug-free medium. Cells were counted every 24 hours. The experiment was performed thrice, and average values are plotted in the graphs. Error bars denote standard deviation. The data for the two HU concentrations tested are presented in separate panels for easier viewing; the 0 mM graph lines are therefore identical in both panels. Raw data excel sheets in Fig. S12.

Short wavelength UV (254 nm) triggers the photochemical fusion of adjacent pyrimidine rings in a DNA strand, leading to the formation of pyrimidine dimers. The cyclobutane rings thus formed cause replication forks to stall. In a two-pronged approach, the cell overcomes this problem by either using a polymerase switch to allow the replication fork to get past the cyclobutane ring or by using the nucleotide excision repair system to repair the damage incurred. The response of *elp3a*-nulls to UV-induced DNA damage (Materials and Methods) was comparable to that of wild-type parasites at the dose tested (Fig. 3A). The topoisomerase I inhibitor CPT primarily targets cells undergoing DNA replication, stabilizing covalent DNA-Topo I complexes and precluding DNA re-ligation, ultimately leading to the accumulation of single-strand breaks (35). In assessing the response of *elp3a*-nulls to doses of CPT ranging from 0 to 25 µM, we found no difference in the behavior of *elp3a*-nulls and *elp3a*^+/+^ cells (Fig. 3B; Fig. S5). The methylation of bases by MMS causes replication forks to stall and eventually collapse, unless repaired. MMS also causes double-strand breaks in non-replicating DNA if the methylated bases are not removed by the base excision repair system. When we compared the behavior of *elp3a*-nulls with that of *elp3a*^+/+^ cells after incubation in medium carrying MMS ranging over 0–0.1%, we found that while *elp3a*^+/+^ and *elp3a*^−/−^ cells were both susceptible to MMS in a dose-dependent manner, *elp3a*^−/−^ cells showed a significantly higher tolerance to the DNA damage-inducing agent (Fig. 3C).

HU is an inhibitor of ribonucleotide reductase, and by depleting the dNTPs pool, it inhibits DNA synthesis, leading to cell cycle arrest. While this is reversible, prolonged exposure to HU leads to collapse of replication forks, coupled to DNA strand breaks. The effect of HU-induced chronic genotoxic stress was assessed by incubating early-log cells (7–9 × 10^6^ cells/mL; Day 3) in HU (0.5 or 1 mM) for 24 hours before washing off the drug (Day 4) and continuing growth in fresh drug-free medium. Upon monitoring cell growth every 24 hours, it was observed that while both cell types were affected by exposure to HU, *elp3a*-nulls recovered rapidly from the treatment and entered a phase of active growth, while *elp3a*^+/+^ cells showed slow recovery from 0.5 mM HU treatment and did not recover from 1 mM HU treatment (Fig. 3D). Taken together, the data in Fig. 3 suggest that deletion of *elp3a* facilitates the cell’s endeavors to overcome MMS-induced and HU-induced genotoxic stress.

### HU-induced chronic stress evokes a differential response on cell cycle progression in Elp3a-depleted cells

**Fig 4:**
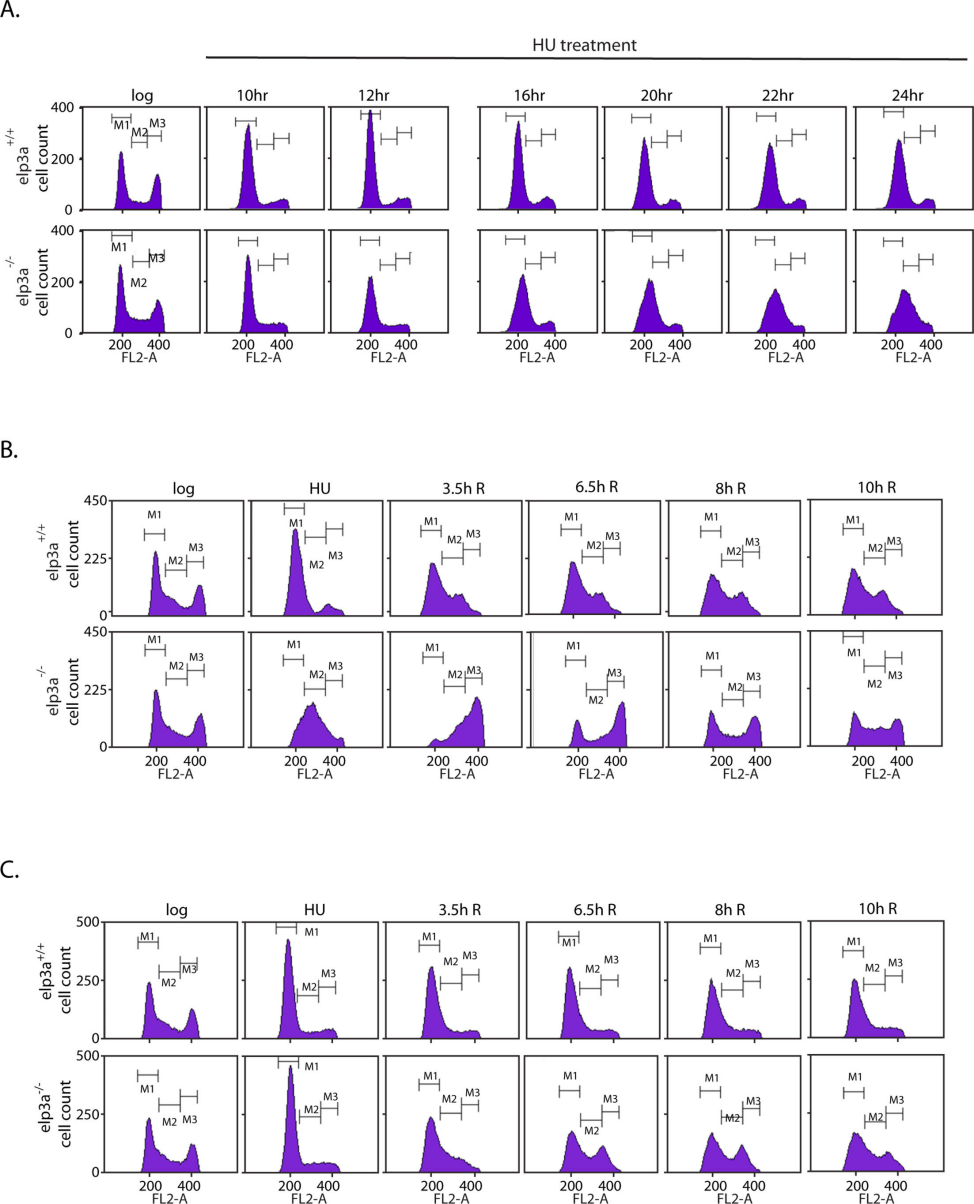
Effect of LdElp3a depletion on the parasite’s response to prolonged HU exposure. (**A**) Analysis of cell cycle progression of parasites devoid of LdElp3a, upon exposure to 1 mM HU for 24 hours. Cells were sampled at various time-points during the 24-hour span period. Sampling time-points are indicated above the histogram panels. Cells in G1, S, and G2M are gated as M1, M2, and M3, respectively. The experiment was done thrice, with comparable results, and one dataset is shown here. (**B**) Analysis of cell cycle progression of parasites depleted of LdElp3a, upon exposure to 1 mM HU for 24 hours followed by washing off the drug and replenishment with fresh medium. Sampling time-points are indicated above the histogram panels. Cells in G1, S, and G2M are gated as M1, M2, and M3, respectively. The experiment was done thrice, with comparable results, and one dataset is shown here. (**C**) Analysis of cell cycle progression of parasites depleted of LdElp3a, upon exposure to 5 mM HU for 24 hours followed by washing off the drug and replenishment with fresh medium. Sampling time-points are indicated above the histogram panels. Cells in G1, S, and G2M are gated as M1, M2, and M3, respectively. The experiment was done thrice, with comparable results, and one dataset is shown here. Gating strategies of experiments of** A, B, and C** are shown in Fig. S15.

Considering the differential responses of *elp3a*^+/+^ and *elp3a*^−/−^ cells to prolonged HU treatment, we analyzed the distribution of cells over the different cell cycle stages while under HU exposure. Accordingly, logarithmically growing promastigotes were incubated in 1 mM HU for 24 hours, and cells were sampled at various times during the incubation period for flow cytometry analysis. It was observed that while *elp3a*^+/+^ promastigotes remained arrested at G1/S throughout the span of incubation in HU, *elp3a*^−/−^ promastigotes behaved differently, being arrested at G1/S up to 12 hours but gradually escaping the arrest thereafter, advancing into S phase (Fig. 4A). In eukaryotes, while several replication origins are licensed, only a fraction fire. The rest remain dormant unless needed, as, for example, when nearby replication forks collapse. The stalling of replication forks in the presence of HU activates the S phase checkpoint and triggers the ATR/Chk1 pathway, which arrests the cell cycle, stabilizes the stalled forks, promotes synthesis of the ribonucleotide reductase subunit to enhance enzyme activity and replace the depleted dNTPs pool, and induces the firing of dormant licensed origins to allow replication to continue (36). We hypothesize that the differential behavior of *elp3a* mutants exposed to HU as compared to wild-type parasites might be due to hyperactivation of the ATR/Chk1 pathway in the mutant cells. Further in-depth investigations will have to be carried out to examine this aspect.

When promastigotes were incubated in 1 mM HU for 24 hours followed by washing off the drug, sampling of cells at various time-points thereafter and analyzing them by flow cytometry revealed that a majority of the *elp3a*^+/+^ cells remained at G1/S or at best moved into early S, and only a fraction of the cells were released into S phase, with this population not reaching G2/M even 10 hours after release from HU (Fig. 4B). Contrastingly, *elp3a*^−/−^ cells, which were already in S phase after 24 hours in HU, navigated S phase to reach G2/M by 3.5 hours after release from HU, continuing to return into G1 thereafter (Fig. 4B). The distribution of cells over the different cell cycle stages while under prolonged exposure to higher concentrations of HU was analyzed by incubating logarithmically growing promastigotes with 5 mM HU for 24 hours, before washing off the drug and monitoring cell cycle progression at various times thereafter. As seen in Fig. 4C, while *elp3a*^+/+^ cells remained arrested even after washing off the drug, a fraction of *elp3a*^−/−^ cells were released from G1/S, although these cells did not reach G2/M even 10 hours after release from HU. Collectively, the data in Fig. 4 underlined the fact that *elp3a*^−/−^ cells were better equipped to tolerate HU-induced chronic stress.

### Elp3a depletion causes the parasite to exhibit a deviant DNA damage response upon exposure to HU-induced chronic stress

As prolonged treatment with HU results in a collapse of stalled replication forks leading to DNA strand breaks that must be repaired before DNA replication can resume, as well as causes production of reactive oxygen species (ROS) that attack DNA and lead to double-strand breaks, we investigated if the differential response of Elp3a-depleted cells was due to a hyperactive DNA damage response or more efficient repair in these cells. In trypanosomatids, HR-mediated repair is the primary mode for restoring double-strand breaks (37). We therefore assessed the activation timing of the HR-based repair system following HU exposure, using RAD51 as a marker.

**Fig 5:**
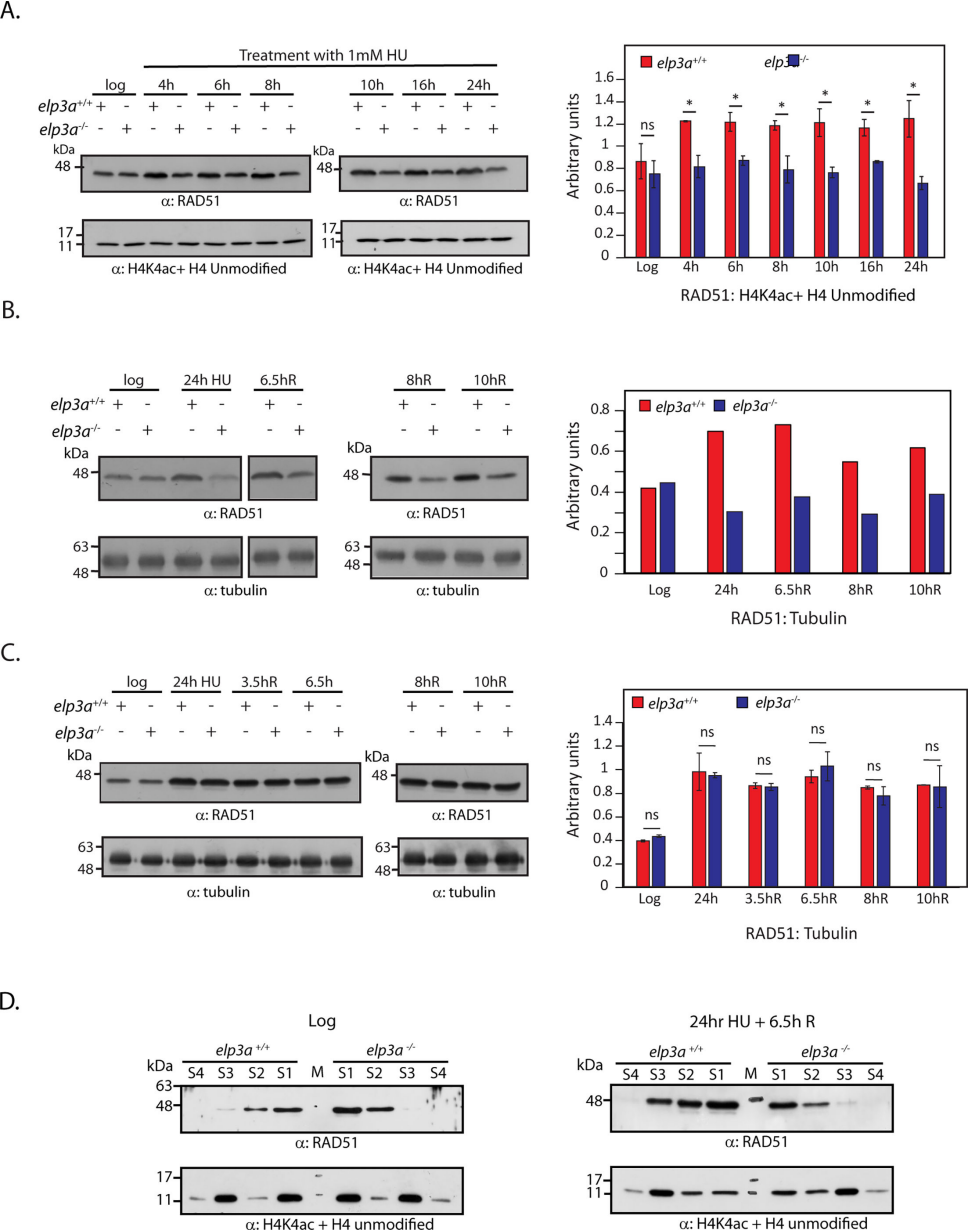
Impact of Elp3a depletion on HU-induced RAD51 activation. (**A**) left panel: Western blot analysis of whole cell lysates isolated from cells that were exposed to HU (1 mM) for the times indicated above the panels, probed with anti-RAD51 antibody (1:1,000 dil). Loading control: anti-H4acetylK4 + anti-H4 unmodified antibodies (1:1,000 dil). Right panel: quantification of blots using ImageJ analysis. Plotted values represent average of two experiments. Error bars indicate standard deviation, and statistical significance was determined using student’s two-tailed *t*-test. *: *P* < 0.05. ns: not significant. Raw data of blot quantifications in Fig. S13. (**B**) Left panel: Western blot analysis of whole cell lysates isolated from cells that were exposed to HU (1 mM) for 24 hours before washing off the drug and continuing incubation in fresh medium for varying times (indicated above the panels). Blots were probed with anti-RAD51 antibody (1:1,000 dil). Tubulin served as loading control. Right panel: quantification of blots using ImageJ analysis. Raw data of blot quantifications in Fig. S13. (**C**) Left panel: Western blot analysis of whole cell lysates isolated from cells that were exposed to HU (5 mM) for 24 hours before washing off the drug and continuing incubation in fresh medium for varying times (indicated above the panels). Blots were probed with anti-RAD51 antibody (1:1,000 dil). Tubulin served as loading control. Right panel: quantification of blots using ImageJ analysis. Plotted values represent average of two experiments. Error bars indicate standard deviation, and statistical significance was determined using student’s two-tailed *t*-test. ns: not significant. Raw data of blot quantifications in Fig. S13. (**D**) Left panel: Western blot analysis of soluble (S1 and S2) and chromatin-bound (S3 AND S4) fractions isolated from logarithmically growing untreated cells. Upper: probed with anti-RAD51 antibodies. Lower: probed with anti-H4acetylK4 + anti-H4 unmodified antibodies to confirm proper fractionation of extracts. Right panel: Western blot analysis of soluble (S1 and S2) and chromatin-bound (S3 and S4) fractions isolated from cells that had been exposed to 1 mM HU for 24 hours and then incubated in drug-free medium for 6.5 hours. Upper: probed with anti-RAD51 antibodies. Lower: probed with anti-H4acetylK4 + anti-H4 unmodified antibodies to confirm proper fractionation of extracts. The experiment was done thrice with comparable results.

To analyze the expression of RAD51 under prolonged HU treatment, logarithmically growing promastigotes (*elp3a^+/+^* and *elp3a^−/−^*) were treated with 1 mM HU for up to 24 hours and analyzed at various times during the incubation period. Parallel cultures that were not subjected to HU treatment were set up as controls. At each time-point of analysis, whole cell lysates were isolated from both untreated and HU-treated parasites and evaluated for RAD51 expression using α-RAD51 antibodies already available in the lab (22). It was found that while RAD51 expression was comparable in untreated logarithmically growing cells of both parasite types (*elp3a^+/+^* and *elp3a^−/−^*), a modest increase in RAD51 expression was detectable in *elp3a^+/+^* cells upon HU exposure as early as 4 hours into HU treatment, and this was sustained till 24 hours of treatment. However, in the case of *elp3a^−/−^* cells, no difference in RAD51 expression levels was observed during HU treatment at any of the time-points analyzed (Fig. 5A). When RAD51 expression levels were similarly assessed in cells that had been incubated in HU (1 mM) for 24 hours and then released into drug-free medium, it was observed that the modest increase in RAD51 expression observed in *elp3a^+/+^* cells after 24 hours of HU treatment was sustained up to 10 hours after washing off the drug (Fig. 5B), suggesting that the incurred DNA damage was not yet completely rectified, an inference that was supported by the cell cycle progression pattern as well, where the majority of the *elp3a^+/+^* cells remained arrested at G1/early S (Fig. 4B). However, contrary to our hypothesis that *elp3a^−/−^* parasites may exhibit a heightened damage response to prolonged HU exposure resulting in hyperactive repair, thus allowing cells to smoothly navigate S phase and G2/M, no difference in RAD51 expression levels was observed in response to HU treatment in the case of *elp3a^−/−^* cells at any of the sampled time-points (Fig. 5B). Upon analyzing RAD51 levels in cells that were similarly challenged with 5 mM HU and then released from drug treatment, however, it was observed that RAD51 expression levels increased substantially in both *elp3a^+/+^* and *elp3a^−/−^* cells after 24 hours of HU treatment, and these levels were sustained even 10 hours after washing off the drug (Fig. 5C), in synchrony with our findings that both *elp3a^+/+^* and *elp3a^−/−^* cells were unable to navigate S and G2/M phases after prolonged treatment with 5 mM HU (Fig. 4C).

The enhanced expression of RAD51 in response to HU-induced chronic stress suggested activation of the HR repair pathway. This pathway involves the formation of RAD51 nucleoprotein filaments, where the single-stranded DNA overhangs at break sites are coated with RAD51 before homology-based DNA repair. As HR-based repair is linked to the loading of RAD51 on chromatin to facilitate strand exchange, we explored the extent of RAD51 loading on chromatin in response to HU-induced chronic stress in Elp3a-depleted cells compared to wild-type cells. To analyze chromatin loading of RAD51, we carried out a fractionation assay (described in Materials and Methods). Briefly, HU-treated (1 mM HU/24 hours) and untreated cells were permeabilized using Triton X-100 to isolate the soluble protein fractions (S1, S2), and this was followed by isolation of DNA-bound protein fractions after treatment of the remaining insoluble fraction with DNAse I (S3, S4). As seen in Fig. 5D (left panels), the amounts of soluble and chromatin-bound RAD51 were more or less comparable between logarithmically growing *elp3a^+/+^* and *elp3a^−/−^* cells. Upon examining the soluble and chromatin-bound protein fractions after treatment of both the cell types with 1 mM HU for 24 hours, followed by 6.5-hour incubation in drug-free medium, it was observed that now substantial amounts of RAD51 were chromatin-bound in *elp3a^+/+^*cells, while the chromatin-bound RAD51 profile of *elp3a^−/−^* cells was similar to its profile in log cells (Fig. 5D right panels).

Taken together, it is evident from the data in Fig. 4 and 5 that while *elp3a^−/−^* cells are more tolerant to HU-induced chronic stress than wild-type cells (displaying a proficient recovery from 1 mM HU treatment unlike wild-type cells, but vulnerable to prolonged treatment with 5 mM HU), the differential response to HU does not seem to be due to a heightened DNA damage response in *elp3a^−/−^* cells as RAD51 activation is not detectable at any of the various time-points analyzed.

### Elp3a depletion protects the parasite from induction of DNA damage upon prolonged HU exposure

**Fig 6:**
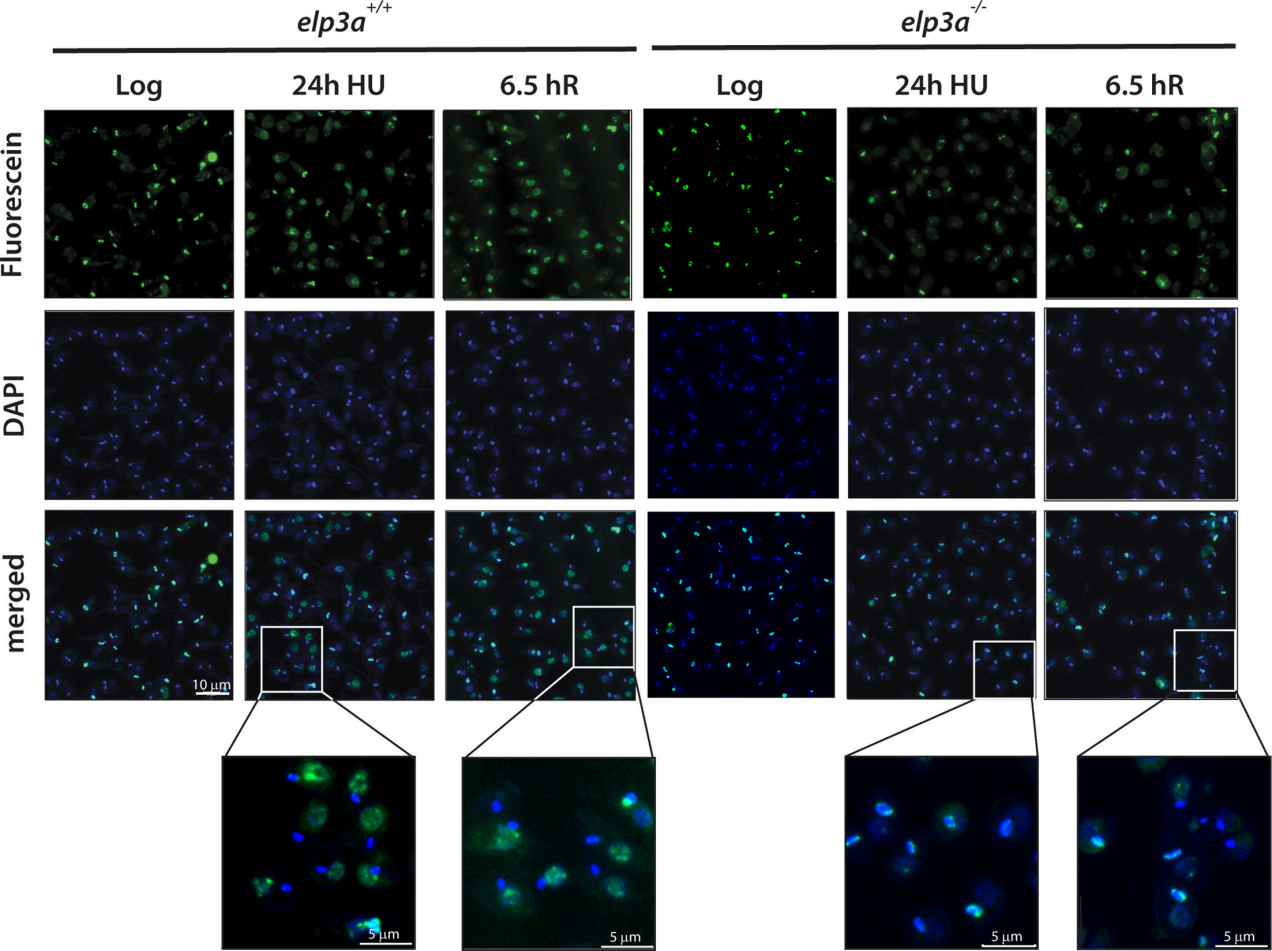
Analysis of DNA damage in Elp3a-depleted cells subjected to prolonged HU exposure. Microscopic analysis of TUNEL assay reactions carried out on cells that were exposed to 1 mM HU for 24 hours. First row: fluorescein-labeled nuclear and kinetoplast DNA. Second row: DAPI-stained nuclear and kinetoplast DNA. Third row: merged image of fluorescein and DAPI. Log column: untreated cells. 24 h HU column: cells incubated in 1 mM HU for 24 hours. 6.5 hR column: cells incubated in 1 mM HU for 24 hours and then in drug-free medium for a further 6.5 hours. Images were captured by Z stack analysis. Magnification bar: 5 µm. The experiment was performed thrice with comparable results.

Failure to detect RAD51 activation in *elp3a^−/−^* cells at any point after prolonged HU treatment led us to consider two possibilities: (i) that an alternate repair pathway was being used and (ii) that the cells were not suffering DNA damage to the same extent in the very first place. We addressed the second possibility first, directly assessing DNA damage in HU-treated cells using the TUNEL assay. This assay is based on the working principle that the enzyme terminal deoxynucleotidyl transferase (TdT) adds fluorescein-12-dUMP at the 3′-OH ends of DNA strands at break sites. Fluorescein-12-dUMP labeled DNA can be directly visualized using fluorescence microscopy. DNA damage in response to HU-induced chronic stress was analyzed in *elp3a^+/+^* and *elp3a^−/−^* cells, after treatment with HU (1 mM/ 24 hours) followed by release into drug-free medium. Microscopic observations revealed that both *elp3a^+/+^* and *elp3a^−/−^* logarithmically growing cells showed comparable kinetoplast and nuclear labeling, with only 1%–2% of the cells showing nuclear labeling and a large number of parasites showing labeled kinetoplast DNA due to dUMP incorporation in replicating kinetoplasts (38). Contrastingly, upon treatment with HU, a large number of nuclei were labeled in *elp3a^+/+^* but not in *elp3a^−/−^* cells (Fig. 6). These data indicate that depletion of LdElp3a reduced the extent of DNA damage caused by HU-induced chronic stress, rather than heightening the response to DNA damage.

### Ectopic expression of Elp3a in *elp3a*-nulls partially rescues the mutant phenotypes

**Fig 7:**
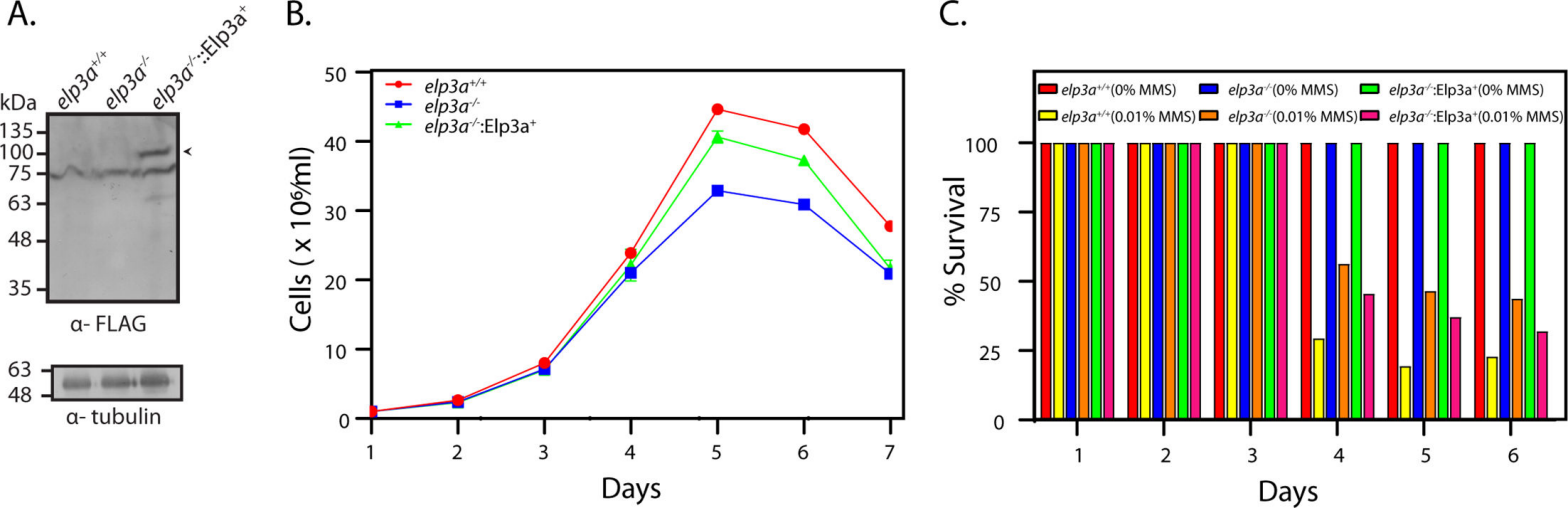
Ectopic expression of Elp3a in *elp3a*-nulls partially rescues the mutant phenotypes**.** (**A**) Western blot analysis of whole cell lysates of transfectant promastigotes expressing Elp3a-FLAG. Tubulin served as loading control. Arrowhead indicates Elp3a-FLAG protein (~85 kDa). (**B**) Analysis of growth of *elp3a*^-/-^ promastigotes expressing Elp3a-FLAG ectopically (*elp3a*^-/-^::Elp3a^+^). Cultures were initiated from stationary phase cultures. The experiment was done twice with technical replicates in each experiment. Values plotted are average of two experiments, and error bars indicate standard deviation. Raw data excel sheets in Fig. S14. (**C**) Analysis of response of *elp3a*^-/-^::Elp3a^+^ promastigotes to MMS exposure. Promastigotes were incubated with 0.01% MMS, and percent survival was determined by dividing the number of cells in the treated culture by the number of cells in the untreated culture, and multiplying by 100. Raw data excel sheet in Fig. S14.

To confirm that the phenotypes observed upon *elp3a* deletion were due to depletion of Elp3a and not due to any secondary mutations elsewhere in the genome, we created a rescue line (Materials and Methods). Accordingly, Elp3a-FLAG was expressed episomally in *elp3a-*null promastigotes to create the line *elp3a^−/−^*::Elp3a^+^ (Fig. 7A), and its growth pattern analyzed in comparison with wild-type and mutant parasites. A partial rescue of the growth phenotype of mutant parasites was observed (Fig. 7B). The response of *elp3a^−/−^*::Elp3a^+^ parasites to MMS exposure was also examined, and here too, a partial rescue of the mutant phenotype was observed (Fig. 7C). While the reason for partial rescue is not known, we speculate that it could be due to differences in Elp3a-FLAG expression levels in the different cells of the population. The rescue of the mutant growth and response to MMS phenotypes by ectopic expression of Elp3a-FLAG indicated that the observed phenotypes of *elp3a*-nulls were not due to secondary mutations elsewhere in the genome.

The data presented above collectively indicate that while LdElp3a is not essential for parasite survival in either promastigotes or amastigotes, it modulates the parasite response to chronic genotoxic stress induced by some DNA damage-inducing agents. The mechanisms by which it may do so await further investigations.

## DISCUSSION

When first identified in *S. cerevisiae* as a complex that co-purified with the hyperphosphorylated form of RNA polII, elongator proteins were believed to play a role in transcriptional elongation (1). While the Elp3 subunit of this ~850 kDa six-subunit complex was identified as the main catalytic subunit, the Elp1–6 holocomplex assembly was needed for efficient catalytic activity (3, 39). Early studies in *S. cerevisiae* found Elp3 to play a role in H3 and H4 acetylations, in modulating telomeric silencing, and in transcriptional elongation (2, 3, 6). Data from subsequent studies, however, suggested that these effects may be indirect, being mediated by Elp3’s role in translation which would impact all of these processes by modulating expression levels of the pertinent proteins (40). While the Elp1–6 holocomplex is found in eukaryotes from yeast to humans, Elp3 is the only elongator protein identified in trypanosomatids so far, with these organisms harboring two Elp3 orthologs: Elp3a and Elp3b. Alsford and Horn (11) studied Elp3 proteins in *T. brucei* and found Elp3b to negatively regulate rDNA transcription. The role of Elp3a has not been uncovered in any trypanosomatid so far, although the same study by Alsford and Horn found *elp3a* to be non-essential to *T. brucei*. The present report discusses the findings of our investigations into the role of one of the Elp3 orthologs of *L. donovani*: LdElp3a.

We found *elp3a* to be non-essential for cell survival in both promastigotes and amastigotes (Fig. 1C and 2). However, the mutant parasites exhibited a differential response to certain genotoxic agents: MMS and HU, as compared to wild-type parasites (Fig. 3C and D), exhibiting a higher tolerance to chronic HU-induced stress (Fig. 4). This was due to a muted response to the damage-inducing agents rather than a hyperactive DNA damage response/repair (Fig. 5 and 6). In attempting to uncover how *elp3a* mutant parasites were being protected from the effects of HU exposure, DNA microarray analysis was carried out (data not shown). Elp3a depletion did not have any significant impact on global gene expression under normal or genotoxic conditions, and no clues as to how LdElp3a was modulating the parasite response to HU exposure were discernible.

**Fig 8:**
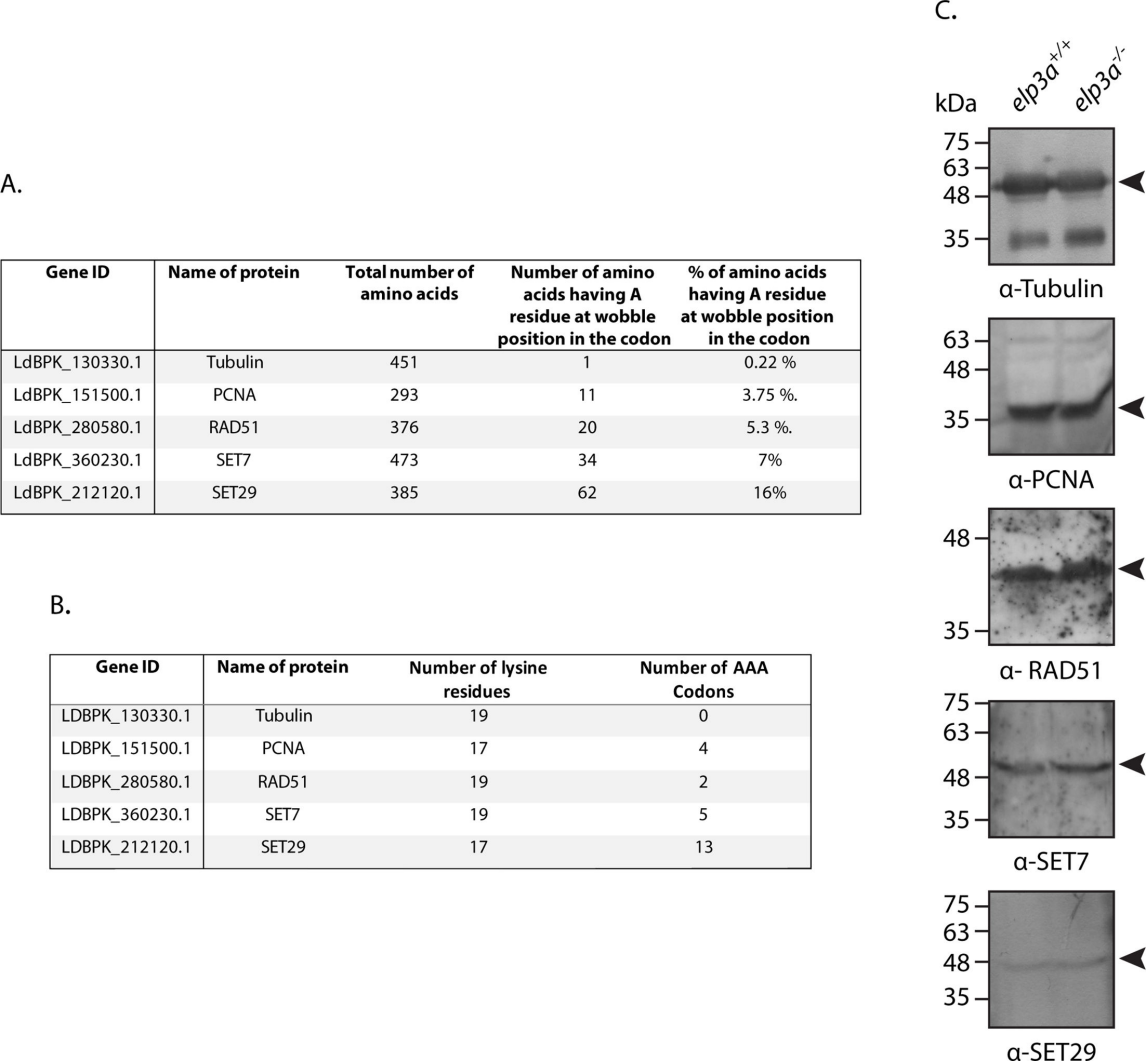
Analysis of impact of Elp3a depletion on translation of proteins rich in codons carrying A residues at the wobble position**.** (**A**) Analysis of protein sequences for the number of amino acids whose codons harbor an A residue at the wobble position. (**B**) Analysis of protein sequences for the number of lysines encoded by the AAA codon. (**C**) Western blot analyses of the proteins listed in **A and B**, in lysates isolated from Elp3a-depleted cells as compared to wild-type cells. Full-length uncropped blots in Fig. S6.

Although the yeast Elp1–6 complex is believed to play a role in multiple processes such as transcriptional elongation, histone H3 acetylation, and telomeric gene silencing, one school of current thought is that Elp3 mediates its impact on these processes through the modulation of translation of the relevant proteins governing these processes. This belief stems from data that emerged from studies which found the elongator complex is required for 5-methoxycarbonylmethyl and 5-carbamoylmethyl modifications of uridines at tRNA anticodon wobble positions. These modifications promoted effective translation events by stabilizing codon-anticodon interactions: expression levels of proteins enriched in codons carrying an adenine residue at the wobble position were significantly lower in *elp3* mutants, and these defects were rescued by overexpression of the pertinent tRNAs in the mutants (8, 27, 40). However, here too, Elp3 exerted its impact only when part of the Elp1–6 holocomplex. To date, there is no evidence of an elongator complex existing in trypanosomatids. Nevertheless, in looking for prospective mechanisms by which LdElp3a may exercise its effect (modulation of response to exposure to DNA damage-inducing agents), we explored the possibility of this protein affecting the translation of proteins that were rich in codons harboring an adenine at the wobble position. To do this, we first analyzed the nucleotide and amino acid sequences of *L. donovani* proteins against whom antibodies were available in the lab and determined the number of amino acids with adenine residues at the wobble position of their codons (Fig. 8A). Specific attention was directed to lysine residues (Fig. 8B), as previous data from studies with *S. cerevisiae* and *Schizosaccharomyces pombe* have shown that overexpressing tRNA^Lys^_S_^2^_UUU’_ in *elp3* mutants rescues the mutant phenotypes (8). To examine the effect of *elp3a* deletion on expression of these various proteins, whole cell lysates were isolated from *elp3a^+/+^* and *elp3a^−/−^* parasites and analyzed by Western blots (Fig. 8C). Keeping in mind the fact that AAA codon-rich proteins in yeast are downregulated in *elp3* mutants (41, 42), we particularly focused on LdSET29, where 16% of the amino acids carry a wobble A in their codons and 13 of the 17 lysines are coded by AAA. However, we found that *elp3a* deletion did not have any significant impact on the expression levels of any of the analyzed proteins including LdSET29 (Fig. 8C), ruling out the possibility of a reduction in translation efficiency in *elp3a* mutants being the root cause of the high tolerance of these mutants to HU exposure. In light of the fact that to date, there is no evidence to suggest that this mechanism of translation regulation exists in *Leishmania* and other trypanosomatids, or even that these modifications of anticodon wobble uridines occur in these parasites, these findings are not entirely surprising. While the ostensible absence of the Elp1–6 holocomplex indicates that many of the functions of the elongator complex seen in other eukaryotes may not be mediated by the *Leishmania* Elp3a, it is also possible that at least some of these functions are being mediated by LdElp3b.

As no reduction in translation efficiency was detectable in *elp3a* mutants, further investigations need to be carried out to determine the cause of the high tolerance of these mutants to HU exposure. Prolonged exposure to HU leads to the production of ROS and RNS. Levels of ROS and RNS produced in *elp3a* mutant promastigotes upon HU exposure would have to be compared with those produced in wild-type parasites, and if found to be significantly lower, this could be due to hyperactive scavenging systems in the mutant. These aspects need further in-depth investigations. The data demonstrates that LdElp3a regulates the parasite response to certain (but not all) genotoxic agents in *in vitro* promastigote cultures, but seemingly contrarily, no difference in infection and survival of *elp3a* mutant in the mammalian host cell is evident, although the intracellular environment of host macrophages would also be genotoxic due to ROS and RNS. While the reason for this remains unknown at this point in time, it appears that Elp3a’s role in modulating the parasite’s response to genotoxic stress may be restricted to the insect form of the parasite. All our experimentation was performed with the procyclic form of promastigotes. Whether this role of Elp3a is limited to this stage of the parasite or extends to the metacyclic form as well remains to be examined.

The enhanced tolerance to HU-induced genotoxic environment upon *elp3a* deletion suggests that LdElp3a normally plays a role in moderating promastigote propagation under genotoxic stress. While there is no direct evidence pointing to promastigotes being exposed to external genotoxic agents in the insect host, the parasite experiences considerable cellular fluctuations and biochemical changes due to differentiation from amastigotes to procyclic promastigotes in the midgut (and subsequently from procyclics to metacyclics). The transformation from amastigotes to promastigotes is coupled to temperature and pH changes in the environment the parasite is being exposed to. Furthermore, ROS-mediated host immune response may be activated by infective bacteria in the insect gut microbiota. These factors would contribute to creating oxidative stress in the promastigotes, which could lead to oxidation of the bases in DNA as well as DNA strand breaks (14, 43). Through the moderation of the promastigote response to these stresses, LdElp3a may control parasite proliferation rates in the insect midgut and prevent *Leishmania* hyper-propagation, thus helping maintain the balance between host and parasite. In working towards deciphering the mechanism of Elp3a action, further experimentation will also be directed toward analyzing the proteome and metabolome of *elp3a* mutant cells.  

## Data Availability

The sequence of Ld1S *elp3a* has been submitted to GenBank, Accession: PX023932.

## SUPPLEMENTAL MATERIAL

Fig. S7 spectrum.02439-25-s0001.xlsx Supplemental figure 7.

Fig. S8 spectrum.02439-25-s0002.xlsx Supplemental figure 8.

Fig. S9 spectrum.02439-25-s0003.xlsx Supplemental figure 9.

Fig. S10 spectrum.02439-25-s0004.xlsx Supplemental figure 10.

Fig. S11 spectrum.02439-25-s0005.xlsx Supplemental figure 11.

Fig. S12 spectrum.02439-25-s0006.xlsx Supplemental figure 12.

Fig. S13 spectrum.02439-25-s0007.xlsx Supplemental figure 13.

Fig. S14 spectrum.02439-25-s0008.xlsx Supplemental figure 14.

Supplemental material spectrum.02439-25-s0009.pdf Fig. S1 to S6 and S15.

ASM does not own the copyrights to Supplemental Material that may be linked to, or accessed through, an article. The authors have granted ASM a non-exclusive, world-wide license to publish the Supplemental Material files. Please contact the corresponding author directly for reuse.
