# Supplementary material for: Leishmania donovani elongator protein Elp3a plays a crucial role in modulating the parasite response to genotoxic stress
Source: Microbiol Spectr. 2025 Dec 5;14(1):e02439-25. doi: 10.1128/spectrum.02439-25 (PMC12772310; doi:10.1128/spectrum.02439-25)
Supplement: Supplemental material — Fig. S1 to S6 and S15. [file spectrum.02439-25-s0009.pdf]

***Leishmania donovani* elongator protein Elp3a plays a role in modulating the parasite response to genotoxic stress**

Arushi Khanna [1,2], Shilpa Rohra [1] and Swati Saha [1]*

[1]Department of Microbiology
University of Delhi South Campus
New Delhi-110021
INDIA

[2] Current affiliation:
New Delhi Tuberculosis Centre
Delhi-110002
INDIA.

To whom correspondence may be addressed

Running title: *Leishmania donovani* Elp3a protein

Keywords: *Leishmania donovani*, trypanosome, protozoan parasite, elongator, Elp3a, genotoxic stress

Figure S1

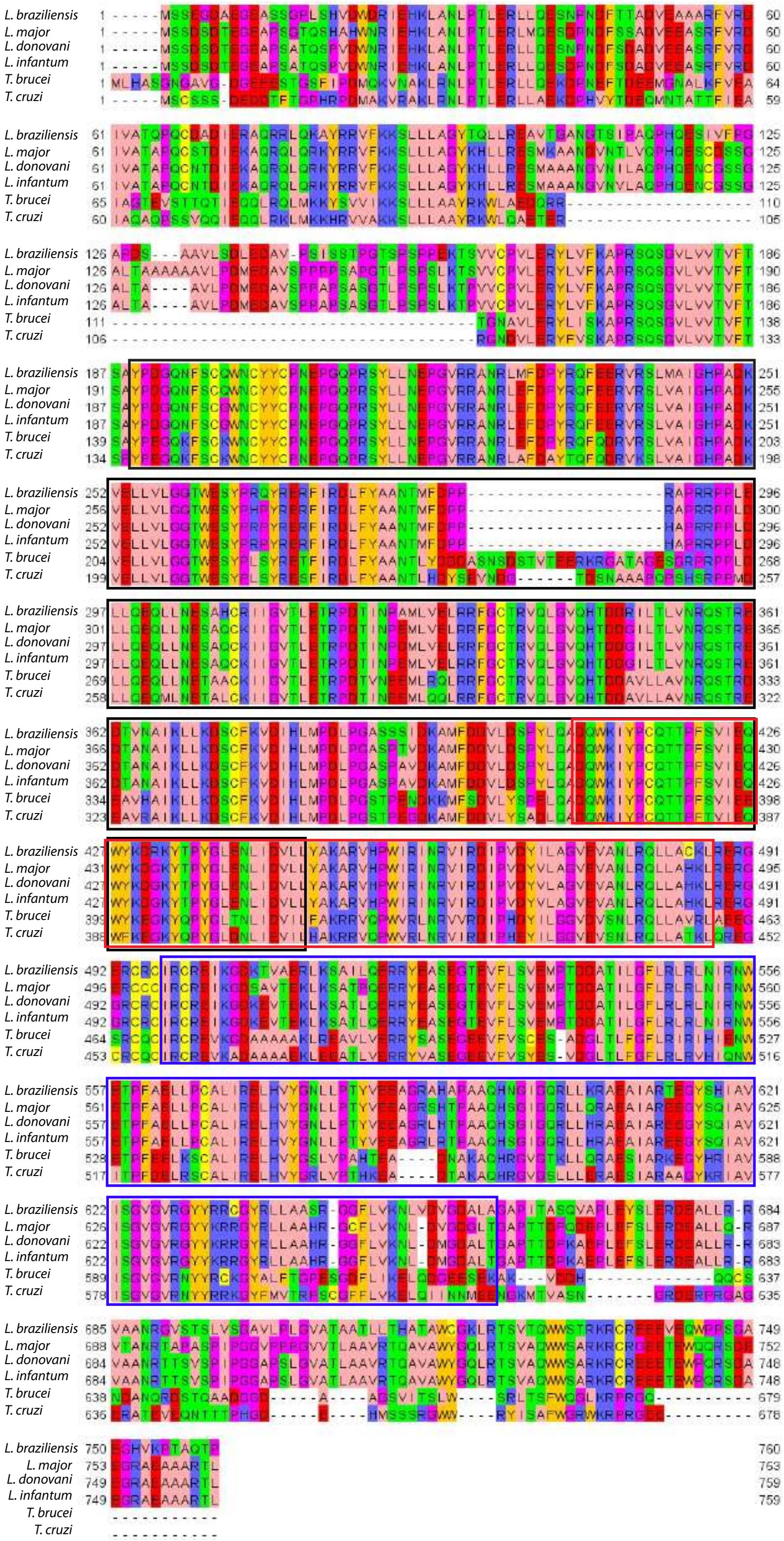

Figure S1: Analyis of LdElp3a amino acid sequence in comparison with those of Elp3a proteins of other trypanosomatids. Clustal Omega analysis (Sievers *et al*., 2011) viewed using Jalview. *L. braziliensis- Leishmania braziliensis*; *L. major- Leishmania major*, *L. donovani- Leishmania donovani 1S, L. infantum- Leishmania infantum, T. brucei- Trypanosoma brucei*, *T. cruzi- Trypanosoma cruzi.* The rSAM core and rSAM C-terminal extension have been demarcated with black and red boxes respectively and the blue box marks the GNAT domain. Different colours represent physi-co-chemical properties of the amino acids- Yellow: cysteine; orange/ochre: aromatic; green: hydrophilic; red: acidic; blue: basic;light pink: proline/glycine; purple: hydrophobic/ aliphatic.Conserved domain are marked with box: rSAM domain: black box, rSAM C-terminal extension: red box; GNAT domain: blue box.

Figure S2

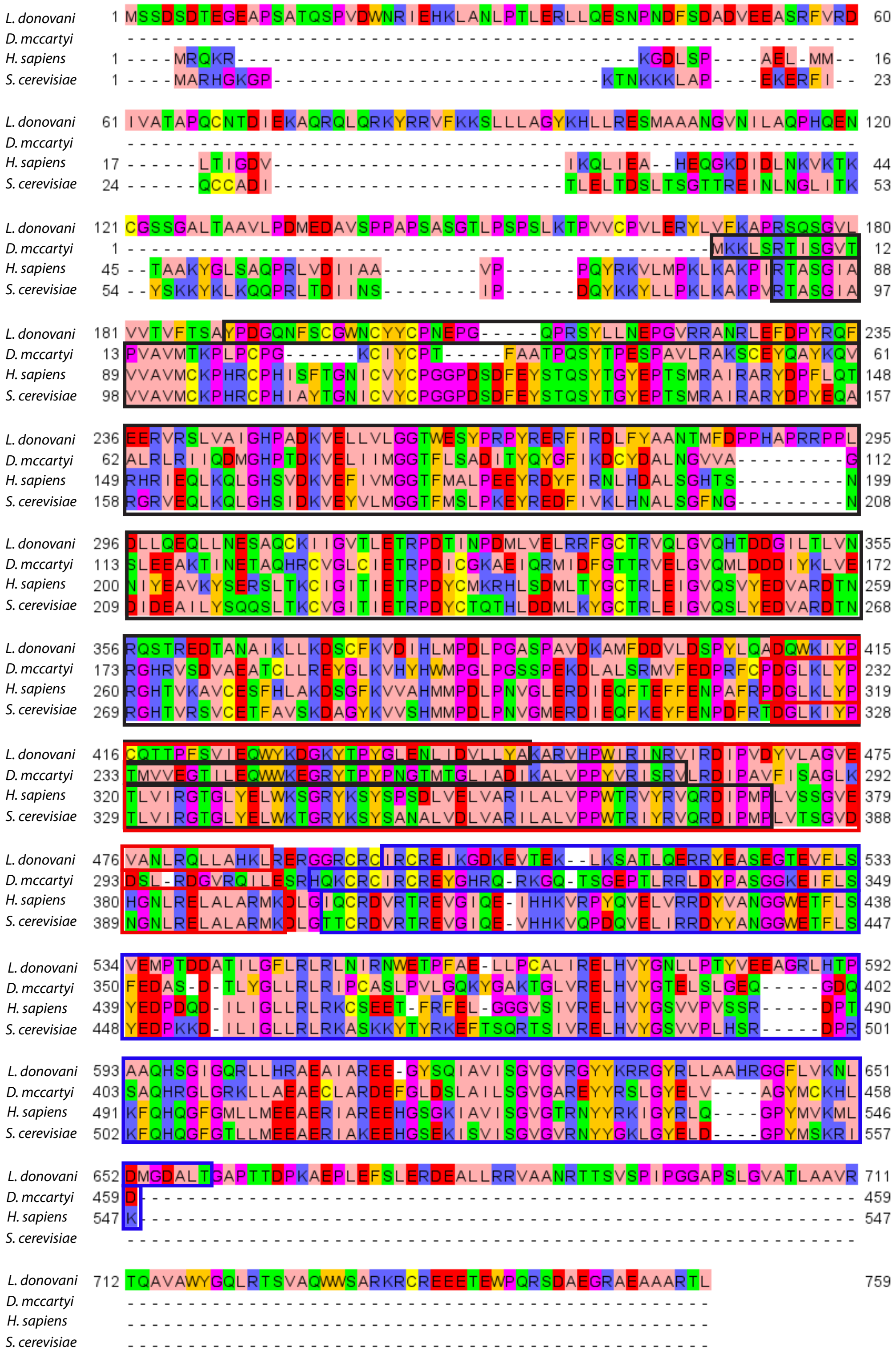

Figure S2: Analyis of LdElp3a amino acid sequence in comparison with those of other eukaryotic Elp3 proteins. Clustal Omega analysis (Sievers *et al.*, 2011) viewed using Jalview. *L. donovani- Leishmania donovani 1S, D. mccartyi- Dehalococcoides mccartyi, H. sapiens- Homo sapiens, S. cerevisiae- Saccharomyces cerevisiae.* Different colours represent physico-chemical properties of the amino acids-Yellow:cysteine; orange/ochre: aromatic; green: hydrophilic; red: acidic; blue: basic; light pink: proline/glycine; purple: hydrophobic/ aliphatic. Conserved domains are marked with box: rSAM domain: black box; rSAM C-terminal extension: red box; GNAT domain: blue box.

Figure S3

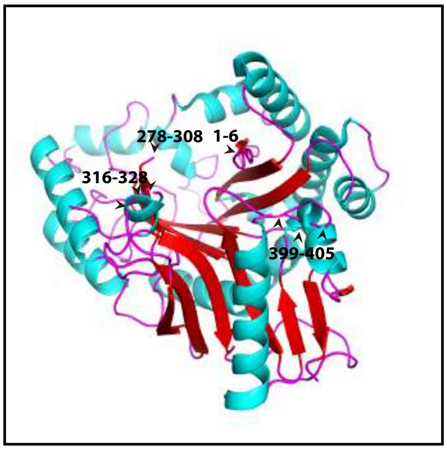

DmcElp3a: PDB ID: 5L7J

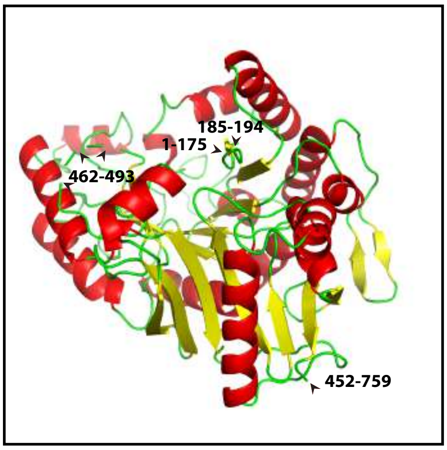

LdElp3a

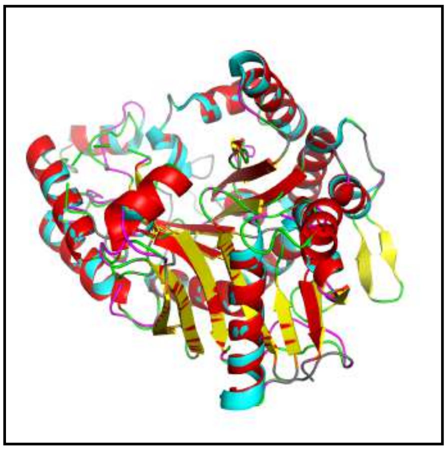

Overlay

Figure S3: ***In silico*** **structural analysis of** ***Leishmania donovani*** **Elp3a:** Upper left panel: *Dehalococcoides mccartyi* Elp3 crystal structure ribbon representation. Cyan represents α-helix, pink represents loop regions and red represent β-sheets. Upper right panel: Ribbon representation of 3D structure of *Leishmania donovani* Elp3a modelled using Phyre2 against DmcElp3 (PDB ID: 5I7J) as template. Red represents α-helix, green represents loop regions and yellow represents β-sheets. Lower panel: View of superimposed structures of LdElp3a and DmcElp3, using PyMOL.

Figure S4

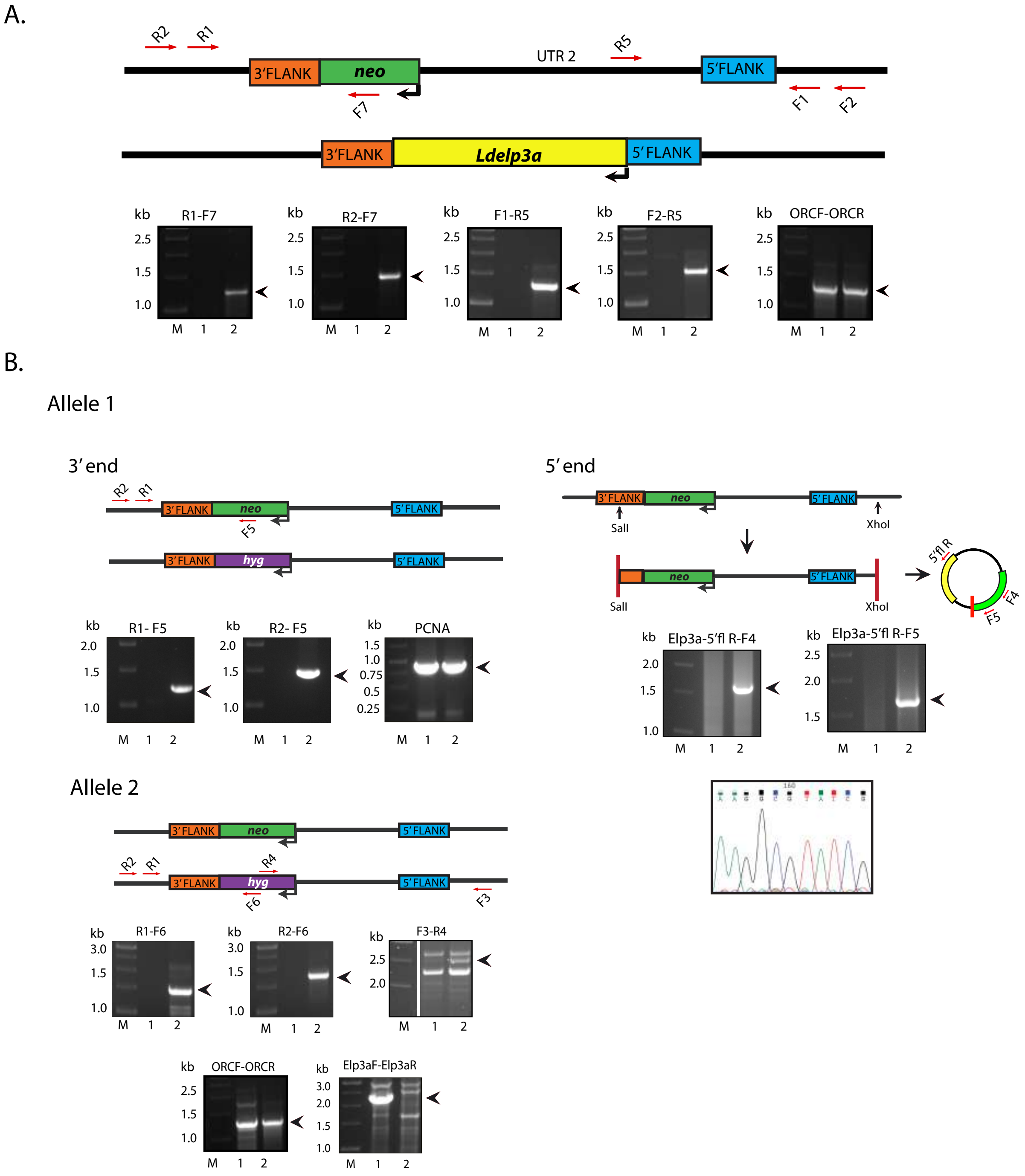

Figure S4: Creation of *elp3a*$^{-/-}$: A. Creation of *elp3a*$^{-/+}$. Authentic homologous recombination at both ends was verified by PCRs across the deletion junctions. Primer positions are indicated on the line diagram with arrows. F1, F2, F7: forward primers. R1, R2, R5: reverse primers. ORC served as input template DNA control. Lanes M: DNA ladder Lanes 1: Ld1S genomic DNA template. Lanes 2: *elp3a*$^{-/+}$ genomic DNA template. Primer pairs used are marked above each agarose gel. Arrows indicate the bands expected. **B.** Authentic recombination at the 3′ end of both genomic alleles were verified by PCRs across the deletion junctions. Authentic recombination at the 5′ end of one of the genomic alleles (replacement with *hyg* cassette) was verified by PCRs across the deletion junction. For ensuring correct recombination at the 5′end of the second allele, inverse PCR was carried out: genomic DNA was digested with XhoI and SalI enzymes (positions marked on the line diagram) and self-ligated before using as template in PCRs. Positions of primers used are indicated with arrows. F3, F4, F5: forward primers. R1, R2, R4, 5′fl-R: reverse primers. PCNA and ORCF-ORCR served as input template DNA controls.

Figure S5

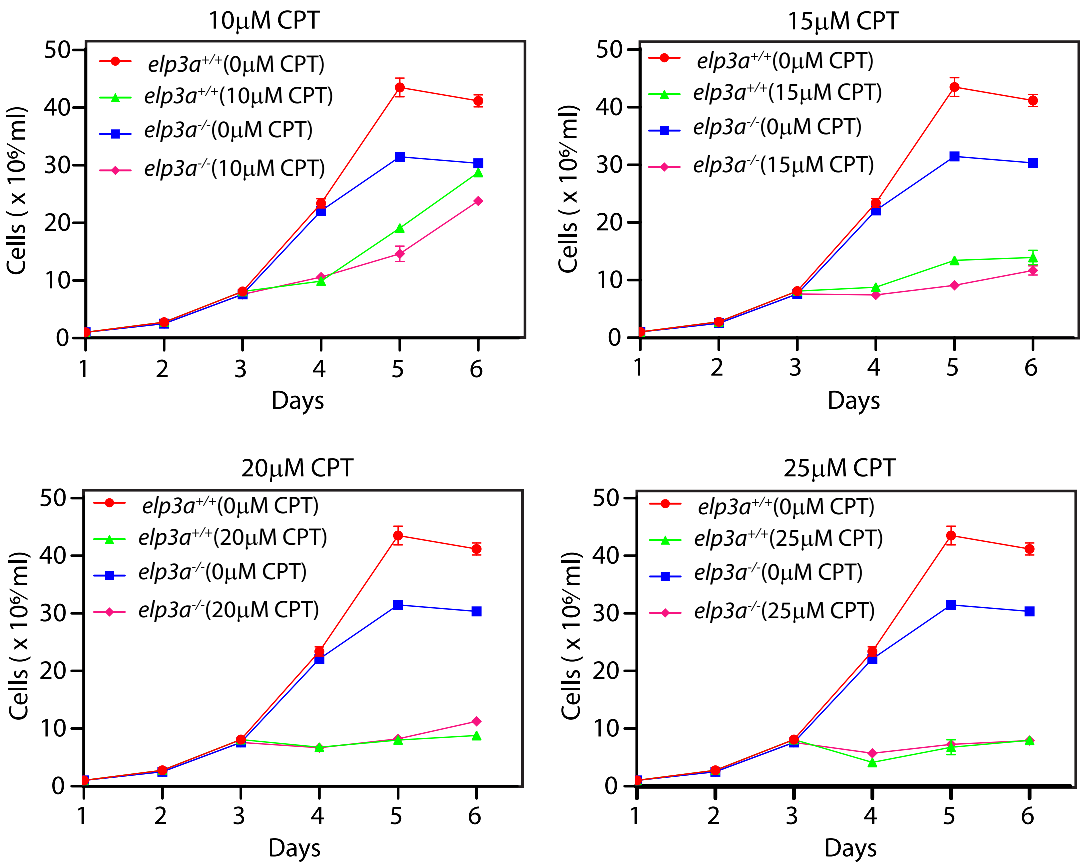

Figure S5: Effect of Elp3a-depletion on the parasite's response to CPT exposure: Cultures were seeded from stationary phase cultures and CPT (10 μM, 15 μM, 20 μM, 25 μM CPT) added after 48 hours. Cells were counted every 24 hours. Values plotted are average of three experiments . Error bars depict standard deviation. The data for each CPT concentration tested is presented in separate panels for easier viewing and thus the 0 μM graph lines are identical across all panels.

Figure S6

Fig 1B.

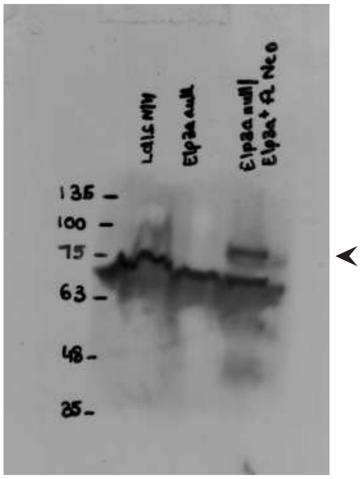

Anti- FLAG

Fig 1B.

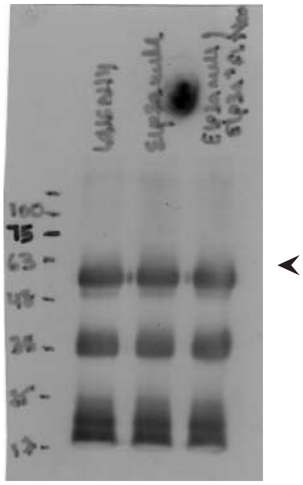

Anti- Tubulin

Fig 7A.

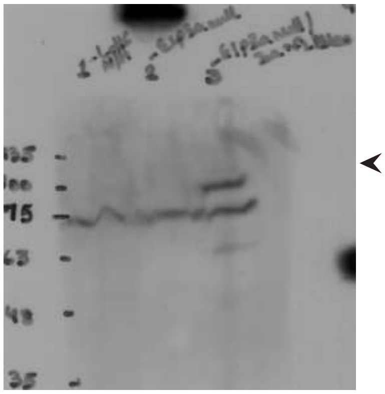

Anti- FLAG

Fig 7A.

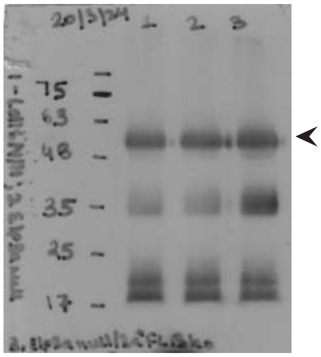

Anti- Tubulin

Fig 8C.

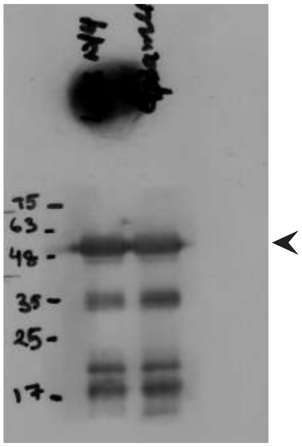

Anti- Tubulin

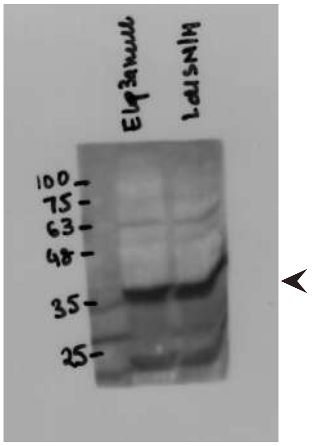

Anti- PCNA

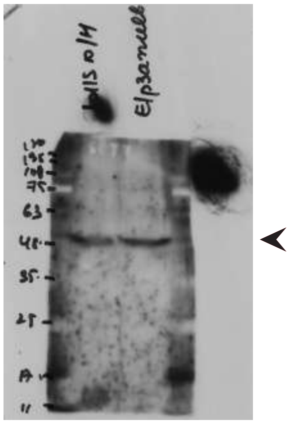

Anti- SET7

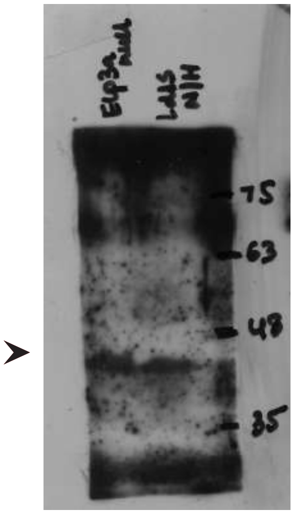

Anti- RAD51

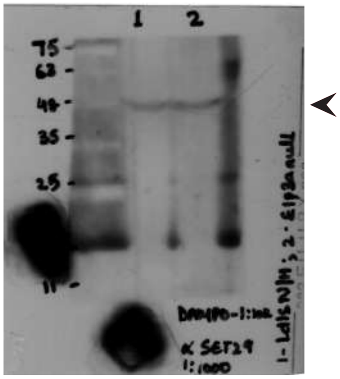

Anti- SET29

Continued overleaf

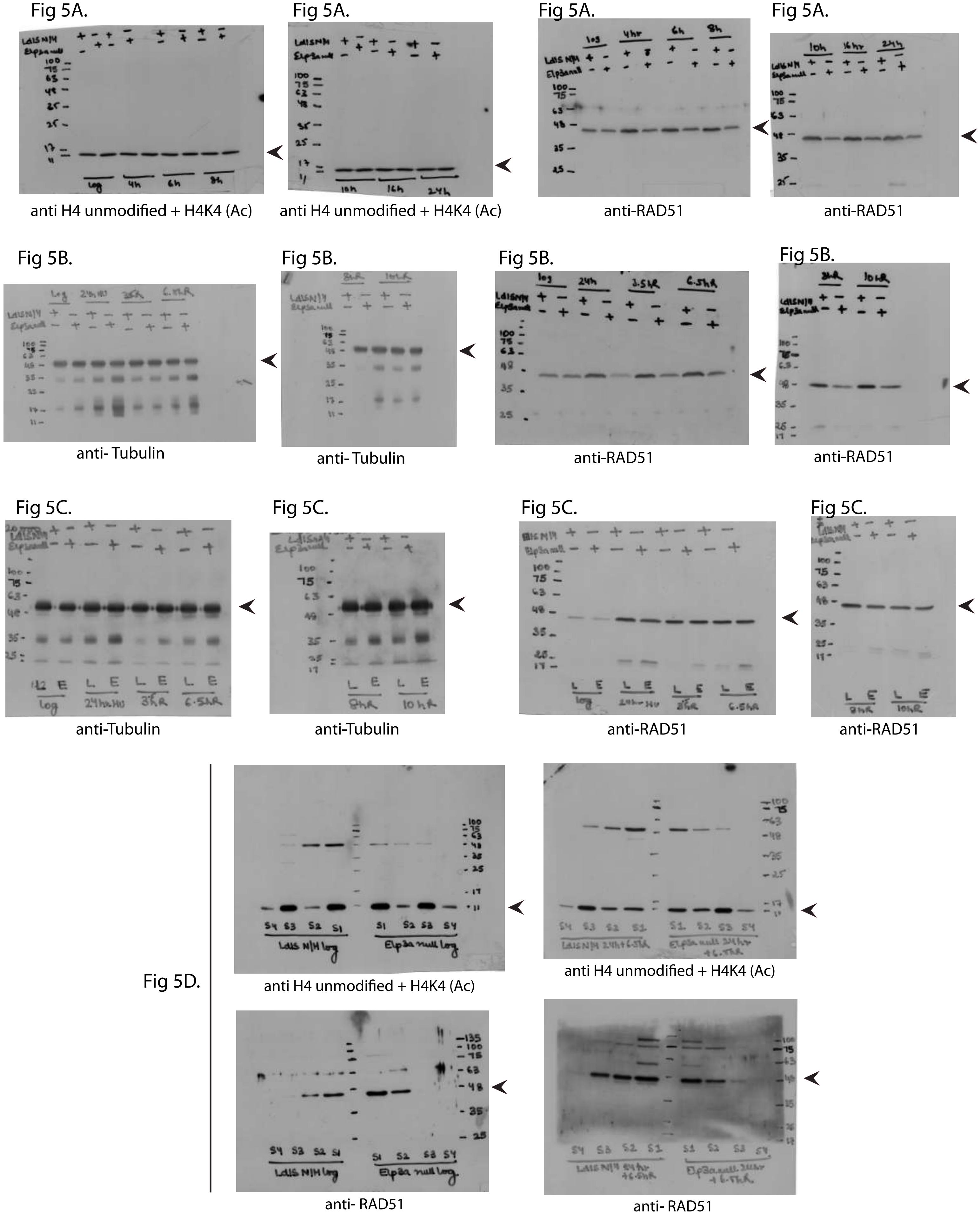

Figure S6 : Uncropped blot images of Figures 1, 5, 7 and 8. Each blot is labelled with the figure number whose uncropped blot it shows.

Figure S15

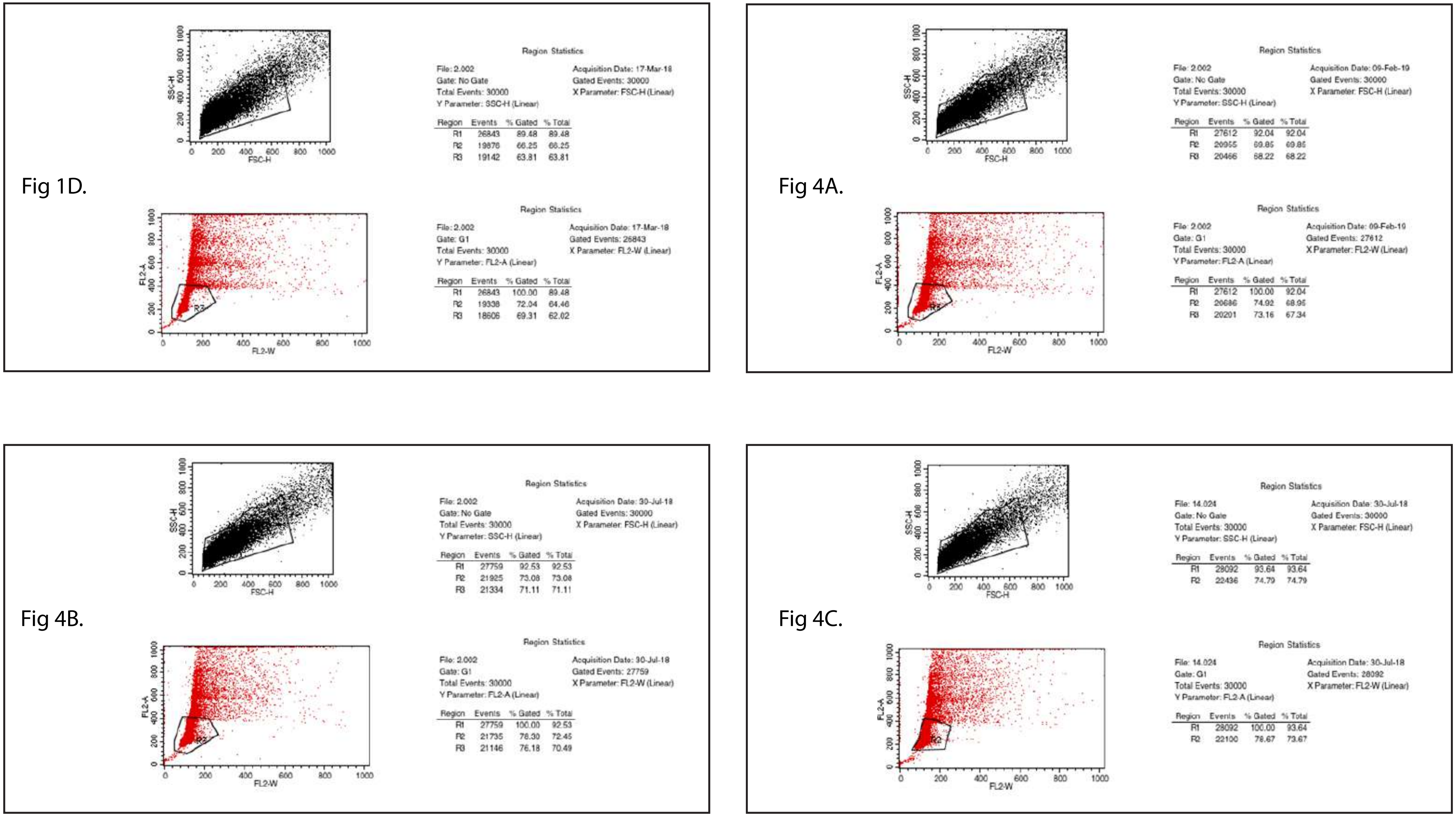

Figure S15: Gating strategies of flow cytometry data of figures 1D and 4A-C: Each panel is labelled with the figure number whose gating strategy it shows.
